# Associations between serum lactate and renal dysfunction in diabetic kidney disease: An exploratory analysis integrating animal experiments, clinical cohorts, and bioinformatic approaches

**DOI:** 10.1371/journal.pone.0358359

**Published:** 2026-09-15

**Authors:** Zongtao Li, Wenyu Zhang, Die Fang, Bingwu Zhao, Tianwei Zhang, Xueqin Zhang, Zhiqiang Chen

**Affiliations:** 1 Hebei University of Chinese Medicine, Shijiazhuang, Hebei, China; 2 First Affiliated Hospital of Hebei University of Traditional Chinese Medicine, Shijiazhuang, Hebei, China; Kwara State University, NIGERIA

## Abstract

**Objectives:**

Diabetic kidney disease (DKD) is a major cause of end-stage renal disease, but the metabolic changes associated with its progression are still not well understood. Lactate, traditionally regarded as a byproduct of glycolysis, has been increasingly recognized in recent studies in association with broader metabolic and regulatory processes. This study aimed to evaluate the association between serum lactate and renal dysfunction in DKD and to explore candidate molecular pathways linking lactate-associated metabolic alterations to DKD-related renal injury using animal experiments, clinical cohort analyses, and exploratory bioinformatic approaches.

**Methods:**

We established DKD in rats by combining right nephrectomy with streptozotocin-induced diabetes to examine metabolic and kidney changes. Clinical associations were evaluated using data from 593 DKD patients in the MIMIC-IV database and were further examined in an independent eICU validation cohort. Associations between serum lactate and renal function were analyzed using correlation and multivariable regression. Candidate molecular pathways were explored using integrated network-based bioinformatics, including target prediction, protein–protein interaction analysis, enrichment analysis, and molecular docking.

**Results:**

DKD rats showed significantly elevated serum lactate and evidence of kidney injury, with lactate strongly correlating with the kidney function marker cystatin C (ρ = 0.748, *P* = 0.0081). In clinical data, elevated lactate was independently associated with higher serum creatinine (β = 0.13, 95% CI: 0.004–0.256, *P* = 0.044), and this positive association was further supported in the external eICU cohort. Network analysis identified 43 overlapping targets linking lactate to DKD, highlighting hub proteins such as PTGS2, EGFR, TP53, ESR1, MAPK3, and MMP9, mainly enriched in inflammation, fibrosis, and metabolism-related pathways.

**Conclusions:**

Elevated serum lactate was associated with renal dysfunction in DKD across animal and clinical datasets. In ICU-based clinical cohorts, this association suggests that lactate may reflect concurrent metabolic and hemodynamic stress related to renal dysfunction, rather than serving as a DKD-specific biomarker or causal mediator. Exploratory bioinformatic analyses identified candidate inflammatory, fibrotic, vascular, and metabolic pathways that may provide molecular context for this association. These computational findings should be interpreted as hypothesis-generating and require further experimental validation.

## Introduction

Diabetic kidney disease (DKD) is a major microvascular complication of diabetes and one of the leading causes of end-stage renal disease (ESRD) worldwide [1,2]. The global burden of DKD continues to increase in parallel with the rising prevalence of type 2 diabetes, which accounts for the majority of DKD cases [3]. Despite advances in glucose control and renin–angiotensin system blockade, many patients still experience progressive loss of kidney function [2,3]. These persistent challenges suggest that mechanisms beyond conventional glycemic and hemodynamic pathways, including mitochondrial stress, hypoxia-related glycolytic shifts, inflammatory–fibrotic remodeling, and broader metabolic dysregulation, may contribute to DKD-related renal dysfunction. Therefore, identifying metabolic indicators that reflect these insufficiently targeted processes may help improve risk characterization and generate biologically plausible hypotheses for further mechanistic studies.

Disturbances in renal energy metabolism are a defining feature of DKD [4]. In diabetic kidney disease, renal energy metabolism may shift toward glycolysis, leading to increased lactate production, which may be associated with kidney tissue injury [5]. Lactate, traditionally viewed as a byproduct of anaerobic metabolism, is increasingly recognized as a molecule associated with metabolic stress and broader regulatory processes. For example, lysine lactylation has been reported as one mechanism through which lactate may be linked to gene regulation [6]. However, protein lactylation was not directly assessed in the present study; therefore, this concept is mentioned only as background context rather than as a mechanistic focus of our analysis. In the kidney, altered lactate metabolism has been associated with cellular stress responses, inflammation, and fibrotic remodeling in diabetic contexts [7,8]. Together, these observations suggest that lactate may reflect broader metabolic and regulatory disturbances relevant to DKD [9,10]. However, evidence directly connecting lactate-associated clinical phenotypes with DKD-relevant molecular networks remains fragmented. In particular, few studies have integrated experimental observations, large-scale clinical data, and systems-level in silico analyses within a single framework to evaluate lactate-associated renal dysfunction in DKD.

To address this challenge, we first established a DKD rat model to examine metabolic changes associated with kidney injury, focusing on lactate. Building on these findings, we analyzed clinical data from the MIMIC-IV database [11,12] to determine whether similar lactate–kidney function associations occur in patients.

Finally, we used integrated network-based bioinformatics [13,14], including target prediction, protein–protein interaction analysis, and molecular docking to explore candidate molecular pathways through which lactate-associated metabolic alterations may be linked to DKD-related renal injury. By combining these approaches, we aimed to generate clinically and biologically plausible hypotheses rather than to establish causal mechanisms.

## Materials and methods

### Experimental animals

#### Animal.

All animal procedures were reviewed and approved by the Ethics Committee of Hebei University of Chinese Medicine (Approval No.: DWLL202401020) and were conducted in accordance with institutional guidelines for the care and use of laboratory animals. Twelve male Sprague–Dawley rats (6 weeks old, 180–200 g) were purchased from Beijing Sipeifu Biotechnology Co., Ltd. (License No.: SCXK (Jing) 2019−0004) and housed under specific-pathogen-free conditions (20–25 °C; 50–70% humidity; 12-h light/dark cycle) with ad libitum access to sterilized chow and water.

#### Methods of sacrifice.

At the endpoint, rats were deeply anesthetized with intraperitoneal 1% sodium pentobarbital (45 mg/kg) and euthanized by exsanguination via abdominal aortic blood collection. Cervical dislocation was performed as a secondary physical method to ensure death (performed by trained personnel). Death was confirmed by the absence of spontaneous respiration and heartbeat prior to tissue collection.

#### Methods of anesthesia and analgesia.

Before surgical manipulation, anesthesia was induced by intraperitoneal injection of 1% sodium pentobarbital (45 mg/kg). Postoperative analgesia was provided with ibuprofen (15 mg/kg) once daily for 24 h after surgery.

#### Efforts to alleviate suffering.

Animals were monitored at least once daily for general condition, wound healing, mobility, and food/water intake. Humane endpoints were predefined to minimize suffering; animals showing severe or persistent distress, marked weight loss, inability to access food or water, or severe immobility would be euthanized immediately after detection. No animals met humane endpoint criteria or died unexpectedly during the experiment, and no animals were excluded after group allocation. Outcome measurements and data analyses were performed in a blinded manner.

#### Establishment of the DKD rat model.

Following a week of acclimation, the animals were divided at random into two groups (six rats each) using a random number table: a model group (MG) and a blank control group (BG). MG rats underwent right nephrectomy under aseptic conditions via a right flank incision, whereas BG rats received sham surgery involving a flank incision and exposure of the renal capsule without kidney removal. After a one-week recovery period, MG rats were fasted for 12 h and injected intraperitoneally with streptozotocin (STZ, 40 mg/kg) freshly prepared in 0.1 mol/L citrate buffer (pH 4.5) to induce diabetes [15], whereas BG rats received an equal volume of citrate buffer. Diabetes was confirmed by measuring tail-vein blood glucose using a Yuwell glucometer for three consecutive days after STZ injection; rats with three random glucose values > 16.7 mmol/L were considered diabetic. The unilateral nephrectomy plus STZ-induced diabetes model was selected to generate a DKD-like renal injury phenotype within the experimental timeframe and to provide exploratory animal evidence for the subsequent clinical and bioinformatic analyses. This composite model was not intended to separate the independent effects of nephrectomy and hyperglycemia, and this limitation is addressed in the Discussion.

#### Sample collection and biochemical measurements.

**Fig 1:**
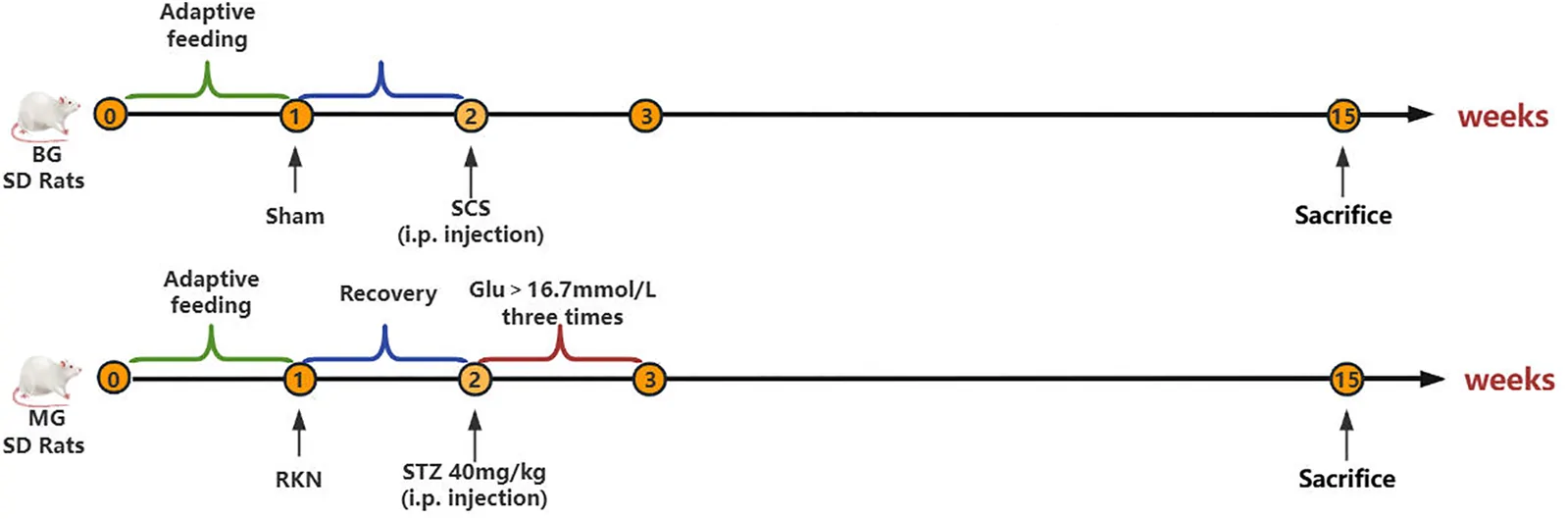
The schematic diagram illustrates the overall workflow of the animal experiment. BG, blank group; MG, model group; GLU, blood glucose; RKN, right kidney nephrectomy; SCS, sodium-citrate solution, citrate buffer; STZ, streptozotocin.

Following 12 weeks of standard feeding, each rat’s body weight was measured. Urine was collected over a 24-hour period in metabolic cages to determine total protein output. After an overnight fast, with water available, anesthesia was induced by intraperitoneal injection of 1% sodium pentobarbital at a dose of 45 mg/kg body weight. Blood was then obtained from the abdominal aorta. The collected samples were spun at 3,000 × *g* for 20 minutes at 4 °C, and the resulting serum was kept at −80 °C until further analysis. A schematic outline of the experimental timeline is presented in Fig 1, including a 1-week acclimation period, sham surgery or right nephrectomy at week 1, intraperitoneal injection of SCS or STZ (40 mg/kg) at week 2, confirmation of hyperglycemia (glucose >16.7 mmol/L on three occasions), a 12-week follow-up period, and terminal sample collection at week 15. Serum and urine biochemical parameters—including cystatin C (Cys C), lactate (Lac), blood urea nitrogen (BUN), 24-h urinary total protein (24-h UTP) and serum creatinine (Scr)—were measured using a fully automated biochemical analyzer according to the manufacturer’s instructions. Serum Cys C was selected as the primary indicator of renal function because it is more sensitive than serum creatinine for detecting early kidney injury, especially in experimental DKD models [16].

#### Renal histopathological staining.

After terminal blood collection, kidney tissues were harvested, fixed in 4% paraformaldehyde, embedded in paraffin, and sectioned. Hematoxylin and eosin (HE), periodic acid–Schiff (PAS), and Masson’s trichrome staining were performed according to standard protocols to assess renal morphological injury, DKD-like glomerular alterations, and collagen deposition/fibrotic remodeling. Representative images were captured under light microscopy.

#### Statistical analysis.

All statistical analyses were performed using SPSS 26.0 (IBM Corp., Armonk, NY, USA). Given the small sample size (n = 6 per group), between-group differences were assessed using the exact, two-tailed Mann–Whitney U test. Where appropriate, effect sizes and 95% confidence intervals were calculated to complement P values. To emphasize effect magnitude and uncertainty in this exploratory setting, between-group effect sizes were additionally summarized as Hodges–Lehmann median differences (Model − Control) and reported in S1 Table. The animal experiment was designed as an exploratory analysis; therefore, a formal power-based sample size calculation was not performed. Group size (n = 6 per group) was determined a priori based on ethical and feasibility considerations and consistency with prior exploratory studies using similar DKD models [17,18]. The association between serum lactate and Cys C was examined using a partial Spearman correlation, controlling for group (BG vs MG), to reduce the influence of between-group separation on correlation estimates. Correlation coefficients (ρ) and bootstrap 95% confidence intervals were reported. All tests were two-sided, and *P* < 0.05 was considered statistically significant.

### Clinical validation with MIMIC-IV

#### Data source and study population.

Clinical information used in this retrospective study was drawn from the MIMIC-IV database (version 3.1). This publicly available resource contains anonymized medical records from patients treated in the emergency and intensive care units of Beth Israel Deaconess Medical Center in Boston, USA, between 2008–2022. Data access was permitted under the Institutional Review Board approval of that center, which exempted the need for individual informed consent. The author completed the official data-use training and obtained certification (ID: 70699642).

**Fig 2:**
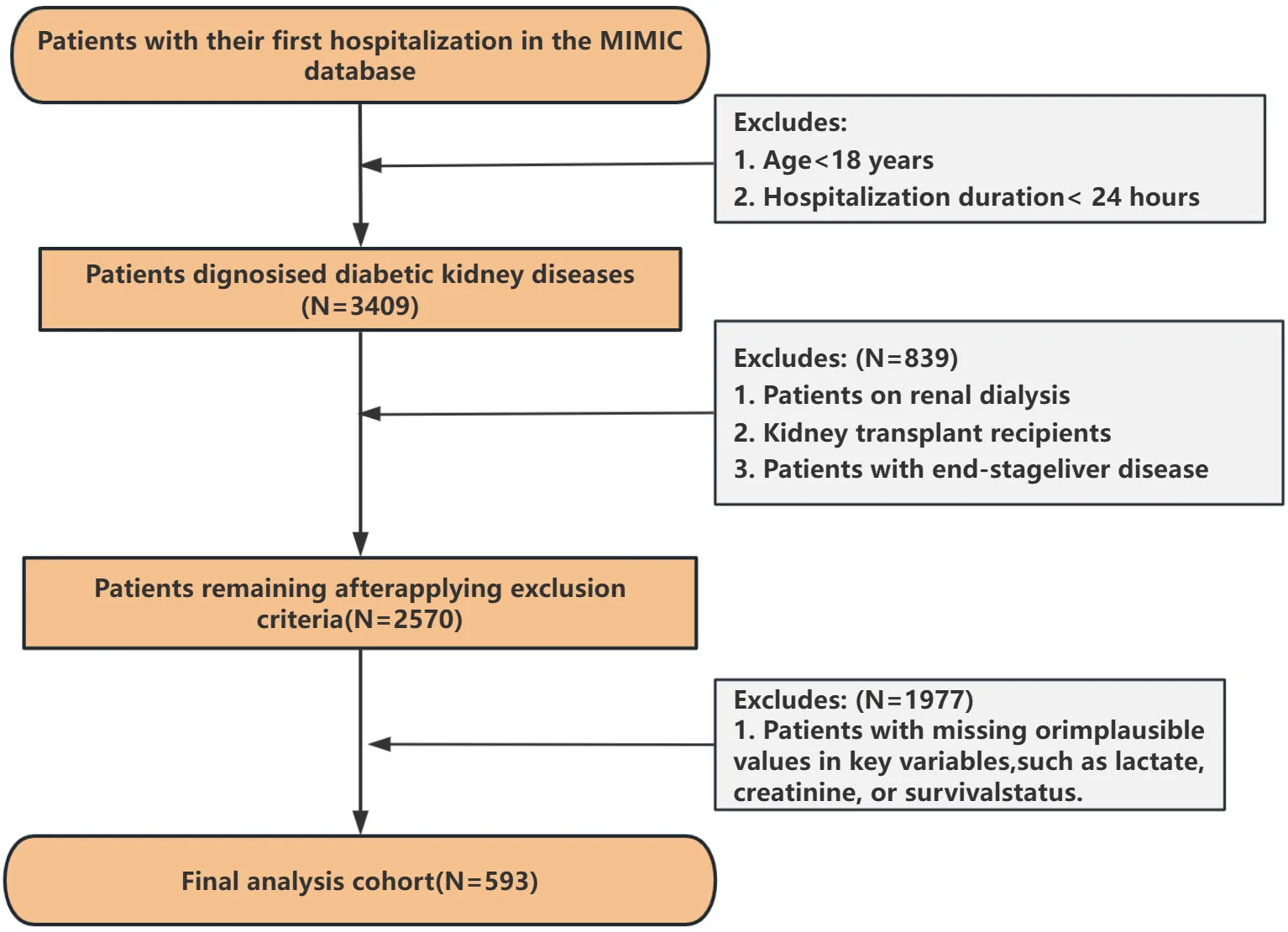
The flowchart illustrates the selection process of DKD patients included in the MIMIC-IV cohort.

MIMIC-IV included 223,453 adult patients whose first hospitalization was documented in the database. DKD cases were identified using International Classification of Diseases (ICD) diagnostic codes. Specifically, ICD-9 codes 249.40, 249.41, 250.40, and 250.42, and ICD-10 codes E08.2, E08.21, E08.22, E08.29; E10.2, E10.21, E10.22, E10.29; E11.2, E11.21, E11.22, E11.29; and E13.2, E13.21, E13.22, E13.29 were used. Patients were classified as having DKD if at least one of these codes was recorded during their first hospitalization. Exclusion criteria were as follows: (1) age < 18 years; (2) hospital stay < 24 hours; for multiple admissions due to DKD, only the first hospitalization was included; (3) kidney transplant recipients, patients undergoing renal dialysis, or those with end-stage liver disease, identified using ICD-9 codes (V42.0, 996.81, V45.1, V45.11, V45.12, V56.0–V56.32, V56.8, 571.2, 571.5, 571.6, 572.2–572.4) and ICD-10 codes (Z94.0, T86.1, T86.10–T86.13, T86.19, Z99.2, Z49.0–Z49.32, K72.0–K72.91, K74.0–K74.69, K76.6, K76.7); and (4) patients with missing values in key variables or values outside prespecified analytic ranges were excluded from the primary analysis (lactate < 0.4 or > 10 mmol/L, serum creatinine < 0.6 mg/dL or > 10 mg/dL, or missing survival status). After applying these inclusion and exclusion criteria, a total of 593 patients with confirmed DKD were retained for the final analysis (Fig 2). Because DKD was identified using ICD coding, some misclassification is possible, and uniform confirmation using albuminuria or longitudinal eGFR data was not feasible for all admissions.

#### Data collection and variable definitions.

We queried MIMIC-IV with SQL, using PostgreSQL 16.6 and working through Navicat Premium 17. The retrieved variables included: (1) Demographic information — age, sex, body weight, and height; (2) Vital signs — systolic blood pressure (SBP), diastolic blood pressure (DBP), mean arterial pressure (MAP), body temperature (TP), heart rate (HR), respiratory rate (RR), Glasgow coma scale (GCS) and sequential organ failure assessment (SOFA); (3) Laboratory indicators — red blood cell count (RBC), white blood cell count (WBC), platelets (PLT), lactate, pH, percutaneous oxygen saturation (SpO_2_), serum creatinine (Scr), blood urea nitrogen (BUN), glucose (GLU), potassium (K⁺), sodium (Na⁺), bicarbonate (HCO_3_^−^), and hemoglobin (HB).

To minimize bias, variables with more than 20% missing values were excluded from the analysis. For variables with missingness below this threshold, multiple imputation was performed in SPSS 26.0 (IBM Corp., Armonk, NY, USA) using the fully conditional specification (FCS) method under the assumption of missing at random. Five imputed datasets were generated, and results were pooled using Rubin’s rules. The following variables were imputed: temperature, heart rate, respiratory rate, and others with missingness less than 20%. Imputation was not applied to variables with no missing data, such as lactate, pH, and others. This approach ensured that the uncertainty introduced by missing values was properly accounted for, thus improving the reliability of our statistical findings. For more details on the variables with missing data and imputation methods, please refer to S2 Table.

The main exposure variable in this study was the earliest lactate value measured within the first 24 hours after hospital admission. The renal outcome was defined as the first serum creatinine value measured after admission, because it was the most consistently available and comparable indicator of baseline kidney function in the MIMIC-IV database. To examine its clinical association, subjects were categorized into three strata according to lactate concentration: low (< 2 mmol/L), moderate (2–4 mmol/L), and high (≥ 4 mmol/L). Renal function served as the outcome indicator and was represented by the first Scr value recorded after admission.

#### Statistical analysis.

Statistical work was carried out in SPSS 26.0 (IBM, Armonk, NY, USA). Data following a normal distribution are reported as mean ± SD and compared with one-way ANOVA followed by Bonferroni adjustment. Non-normal variables are summarized as median and interquartile range (IQR) and tested with the Kruskal–Wallis H method. Categorical results are presented as numbers with percentages and evaluated using the chi-square test. Twenty-eight-day and 365-day mortality were compared across lactate strata using the chi-square test. Normality was checked by the Shapiro–Wilk test when the sample size was ≤ 50, and by the Kolmogorov–Smirnov test for larger samples. Statistical significance was considered at *P* < 0.05 (two-sided).

The association between serum lactate and Scr was evaluated using multivariable linear regression. Four hierarchical models were established: Model 1 was unadjusted; Model 2 was adjusted for sex, age, and BMI; and Model 3 additionally incorporated clinical covariates, including mean arterial pressure, oxygen saturation, and hemoglobin, which were selected a priori as clinically relevant indicators of hemodynamic and oxygen-delivery status; and Model 4 additionally accounted for potential confounding by overall illness severity by further adjusting for the first available SOFA score and GCS score. Because SOFA and GCS had missing values, Model 4 was fitted using a complete-case analysis, and the corresponding sample size is reported. To address potential non-DKD-specific influences on serum lactate, we performed a clinically targeted sensitivity analysis in the MIMIC-IV cohort. This model was designed to partially account for infection- and critical illness-related confounding and adjusted for age, sex, BMI, mean arterial pressure, oxygen saturation, hemoglobin, first available SOFA score, first available GCS score, and sepsis status. These covariates were selected to account for demographic factors, hemodynamic status, oxygenation, oxygen-delivery capacity, overall illness severity, and infection-related critical illness. This analysis was conducted as a complete-case sensitivity analysis because it required the simultaneous availability of all included covariates, and the results are provided in S3 Table. Model assumptions were assessed using standard regression diagnostics, including visual checks of linearity, residual behavior, and influential observations. Subgroup analyses were conducted using the fully adjusted model. Stratified analyses were further performed by sex (male vs. female), age (< 65 vs. ≥ 65 years), and BMI (< 25 vs. ≥ 25 kg/m²). Interaction effects between lactate and stratification variables were examined through multiplicative interaction terms in the regression models. Subgroup analyses were pre-specified and interpreted as exploratory; effect modification was formally assessed using interaction terms, and no additional multiplicity adjustment was applied. Although certain ICU variables such as “vasopressor use” and “mechanical ventilation” exist in MIMIC-IV, their timestamps and dosage details were inconsistently documented, and linkage to the exact time of lactate measurement was not feasible. Including them would introduce additional bias from missingness and temporal misalignment; therefore, these variables were not included in the multivariable regression analyses. To further evaluate the robustness and generalizability of the findings, an external validation analysis was additionally performed in an independent eICU cohort using the same exposure definition, renal outcome, covariates, and hierarchical modeling strategy. Model assumptions were evaluated using standard diagnostic procedures, including visual assessments of linearity, residual behavior checked using Shapiro-Wilk test for normality, and identification of influential observations using Cook’s distance.

#### External validation in eICU.

To externally validate the association observed in the MIMIC-IV cohort, we further analyzed data from the eICU Collaborative Research Database, a large multicenter critical care database in the United States. Inclusion and exclusion criteria for the eICU cohort were defined in the same manner as those used for the MIMIC-IV cohort. The same exposure variable (earliest lactate measured within the first 24 hours after ICU admission), renal outcome (first serum creatinine measured after admission), and hierarchical regression models were applied. Specifically, Model 1 was unadjusted; Model 2 was adjusted for sex, age, and BMI; Model 3 was additionally adjusted for mean arterial pressure, oxygen saturation, and hemoglobin; and Model 4 was further adjusted for SOFA and GCS. To further address potential infection- and critical illness-related confounding, a parallel clinically targeted sensitivity analysis was performed in the eICU cohort by further adjusting Model 4 for sepsis status. Data processing, variable definition, and missing-value handling were performed in the same manner as described above.

### Network pharmacology analysis

#### Collection of lactate structural identifiers and putative targets.

The SMILES, InChI, and SDF formats of lactate were obtained from the PubChem database. Structural identifiers for lactate (SMILES/InChI/SDF) were retrieved from PubChem for downstream computational analyses. Potential lactate-associated protein targets were obtained from STITCH, PharmMapper (fit score ≥ 0.8) and the ChEMBL database. All predicted targets were standardized and deduplicated following UniProt nomenclature rules to generate the final target list. A concise overview of the databases utilized in this workflow is provided in Table 1.

**Table 1:** Databases used in this study.

Database Name	Website
PubChem	https://pubchem.ncbi.nlm.nih.gov/
STITCH	https://stitch.embl.de/
PharmMapper	https://www.lilab-ecust.cn/pharmmapper/index.html
ChEMBL	https://www.ebi.ac.uk/chembl/
UniProt	https://www.uniprot.org/
GeneCards	https://www.genecards.org/
CTD	http://ctdbase.org/
DrugBank	https://go.drugbank.com/
Open Targets	https://www.opentargets.org/
STRING	https://cn.string-db.org/

All databases were accessed between June and August 2025.

#### Screening of DKD-related targets.

Potential DKD-related targets were identified using the keywords “Diabetic Nephropathy” and “Diabetic Kidney Disease” in GeneCards (relevance score ≥ 20), CTD (inference score ≥ 40), DrugBank, and Open Targets (association score ≥ 0.05). The collected gene lists were merged, standardized to official gene symbols, and deduplicated to generate the final set of DKD-related targets. A Venn diagram was used to visualize overlaps among the databases.

The lactate-related targets obtained in the “Collection of lactate structural identifiers and putative targets” subsection were compared with DKD targets in R software (version 4.2.1). Genes that appeared in both sets were regarded as candidate shared targets potentially linking lactate-associated molecular interactions to DKD. These overlapping targets were used for further network analysis. The intersection map was generated using the *VennDiagram* package (version 1.7.3). All databases used in this step are listed in Table 1.

#### Lactate–target–disease network construction and assessment.

The lactate targets and DKD-related targets from the “Screening of DKD-related targets” subsection were loaded into Cytoscape software (version 3.9.1) to build the lactate–target–disease network. Each node in the network represented either lactate or a protein target. Edges indicated their interactions. Network topology was evaluated with the *NetworkAnalyzer* plugin. The connection degree for each node was computed to describe its topological importance within the network structure.

#### Protein-protein interaction network construction and assessment.

Common targets of lactate and DKD were imported into the STRING platform to build a protein–protein interaction (PPI) map limited to *Homo sapiens*. The confidence threshold was defined at 0.4. Interaction results were exported as TSV files and subsequently visualized in Cytoscape. Disconnected nodes were removed before topological analysis. Network characteristics were evaluated using NetworkAnalyzer based on degree centrality, betweenness centrality (BC), and closeness centrality (CC). For module-oriented visualization, PPI nodes were grouped according to functional annotations, including inflammation/ECM remodeling, vascular/neurohormonal signaling, intracellular signaling/stress response, and metabolic/enzymatic or transport-related processes. Node size was scaled according to degree centrality. Key targets were further assessed using the CytoHubba plugin.

#### GO and KEGG enrichment analysis.

Gene Ontology (GO) and Kyoto Encyclopedia of Genes and Genomes (KEGG) pathway enrichment analyses were carried out on the shared targets of lactate and DKD with the R package *clusterProfiler* (version 4.6.2). Pathways or terms with *P* < 0.05 were considered significant. The top 15 GO terms and top 30 KEGG pathways were displayed as bar and bubble plots.

#### Construction of the lactate–target–pathway–DKD network.

A hierarchical “lactate–hub target–pathway–DKD” network was constructed to improve the interpretability of the network-based findings. Representative hub targets were linked to selected enriched pathways and broader biological functions to summarize the putative molecular context connecting lactate-associated targets with DKD-related renal injury. This network was intended to provide a structured overview of database- and enrichment-derived associations rather than to infer experimentally validated sequential regulation.

#### Molecular docking.

Molecular docking was performed to assess the structural plausibility of lactate–protein associations for the prioritized targets. The three-dimensional structure of lactate (SDF format) was downloaded from PubChem, and the 3D structures of the target proteins were obtained from the Protein Data Bank. Protein structures were preprocessed in PyMOL 2.6.0 by removing solvent molecules. AutoDock Tools was then used to add polar hydrogens and assign charges, and both receptor proteins and the lactate ligand were saved in PDBQT format. Docking grid boxes (center and size) were defined using a consistent protocol to cover the predicted binding pocket of each target protein. Docking was carried out with AutoDock Vina v1.2.3, and the resulting poses and docking scores were recorded. The docking conformations and ligand–protein interactions were visualized in PyMOL 2.6.0 and Discovery Studio. Consistent with the hypothesis-generating intent of the in silico workflow, docking results were interpreted qualitatively and were not used to infer quantitative binding affinities or functional modulation.

To enhance methodological robustness and reproducibility, key parameters and thresholds for target screening, network construction, enrichment analyses, and molecular docking were pre-specified and reported. Candidate targets were screened using multiple databases, standardized according to UniProt nomenclature, and further evaluated by PPI network topology, hub-target ranking, GO/KEGG enrichment analysis, and molecular docking. These procedures were used as cross-checking steps to identify biologically plausible candidate pathways rather than as experimental validation. Enrichment results were interpreted at the pathway level rather than as causal evidence. Molecular docking was used only to assess structural plausibility of ligand–protein association; docking scores were not interpreted as quantitative binding affinities, biological activity, or in vivo effect sizes. All in silico analyses were therefore considered exploratory and hypothesis-generating and were interpreted in conjunction with experimental and clinical observations.

## Results

### Elevated lactate levels are associated with renal dysfunction in DKD rats

**Fig 3:**
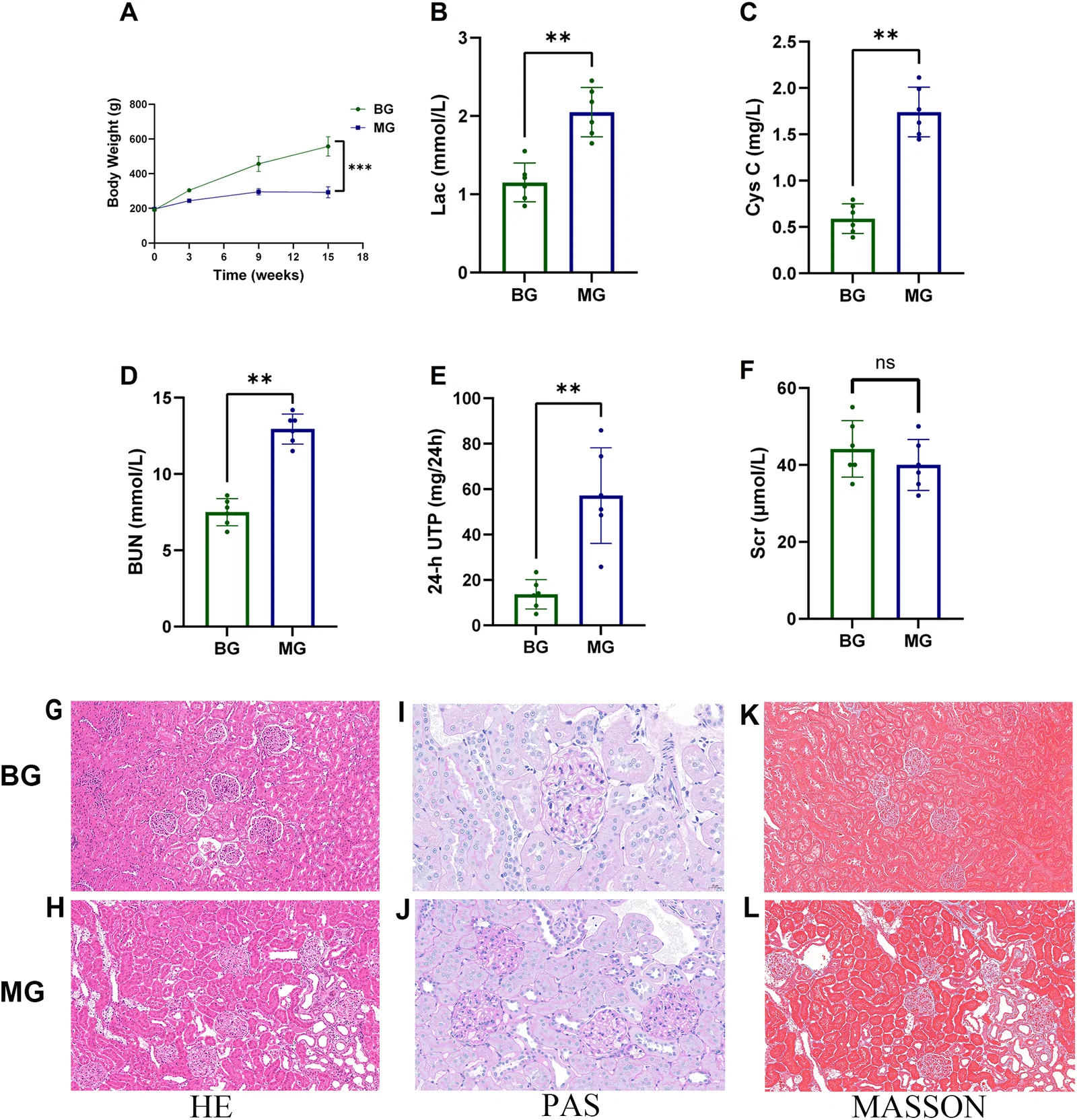
Biochemical and histopathological characterization of the DKD rat model. (A) Body weight. (B) Serum lactate. (C) Cystatin C. (D) BUN. (E) 24-h urinary total protein. (F) Scr. (G–H) HE staining, × 200. (I–J) PAS staining, × 400. (K–L) Masson staining, × 200. *P < 0.05, **P < 0.01, ***P < 0.001; ns, not significant. HE, hematoxylin and eosin; PAS, periodic acid–Schiff.

**Fig 4:**
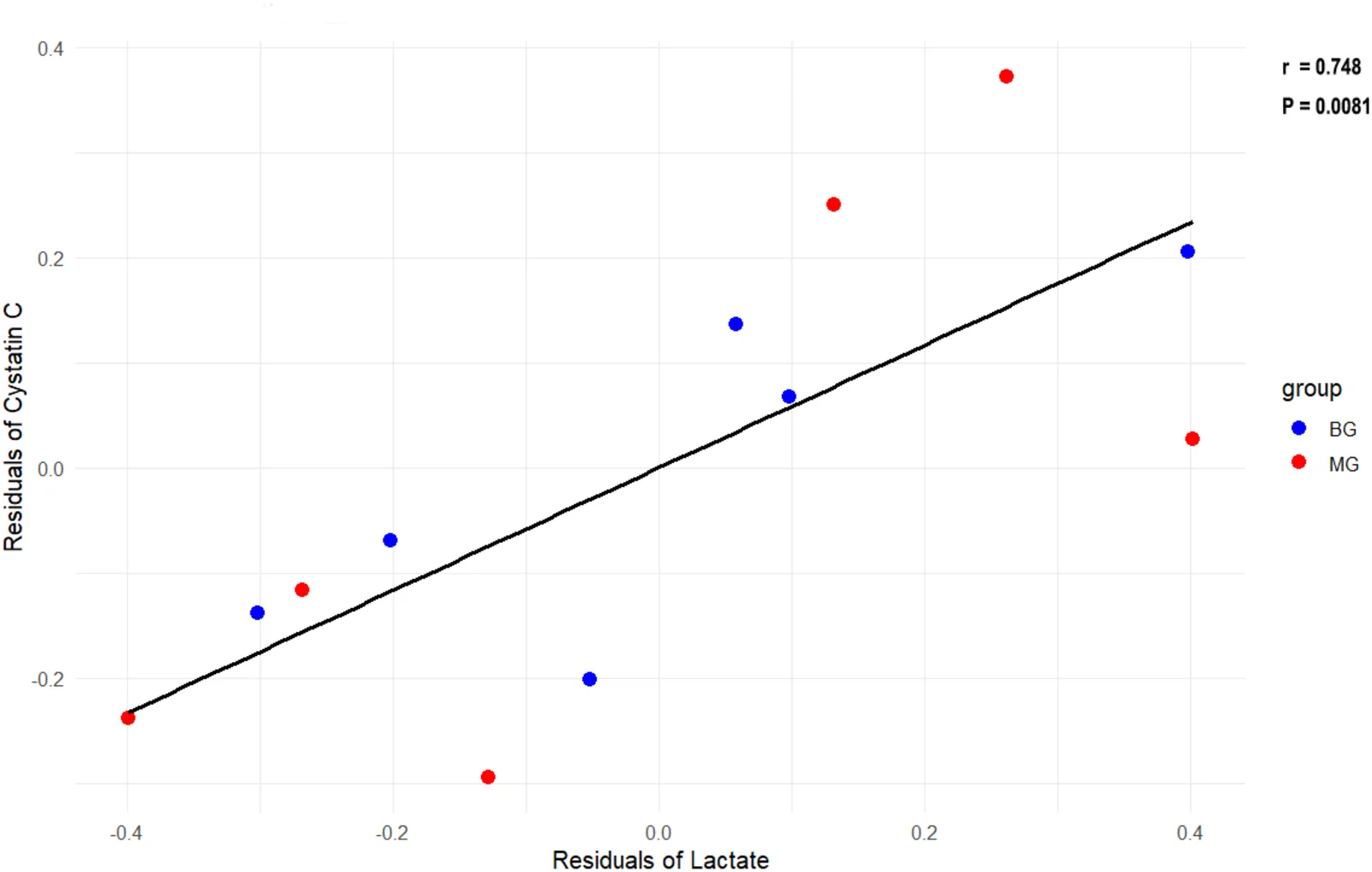
The partial correlation analysis shows a significant positive association between serum lactate and cystatin C after adjusting for group assignment.

As shown in Fig 3, compared with the BG, MG rats showed markedly higher serum lactate levels, accompanied by worse renal function markers reflected by increased Cys C, BUN, and 24-h UTP (P < 0.05), as well as reduced body weight. The difference in Scr was not statistically significant. Histopathological staining further supported renal injury in the MG rats. HE staining showed renal structural alterations in the model group; PAS staining showed DKD-like glomerular changes with increased PAS-positive matrix deposition; and Masson staining indicated enhanced collagen deposition/fibrotic remodeling compared with the BG. Partial Spearman correlation analysis, controlling for group (BG vs MG), showed a positive association between lactate and Cys C (ρ = 0.748, *P* = 0.0081), and should be interpreted cautiously given the small sample size and potential between-group separation (Fig 4). To emphasize effect magnitude and uncertainty given the small sample size, we additionally summarized between-group effect sizes using Hodges–Lehmann median differences (Model − Control) (S1 Table).

After observing an exploratory association between lactate and renal injury markers in the animal model, we next sought to determine its clinical relevance. We therefore analyzed the MIMIC-IV database to verify whether this relationship holds true in a large cohort of ill patients with DKD.

### Clinical validation: Independent association between lactate and Scr in the MIMIC-IV database

#### Baseline characteristics.

A total of 593 DKD patients were analyzed. Among them, 405 (68.3%) were male and 188 (31.7%) were female. The median age was 72 years (IQR 65–80), and the median serum lactate concentration was 1.6 mmol/L (IQR 1.2–2.4). Based on initial lactate levels, patients were stratified into three categories: low (< 2 mmol/L), moderate (≥ 2 and < 4 mmol/L), and high (≥ 4 mmol/L). Baseline characteristics among these groups were compared using appropriate statistical tests (Table 2). No significant differences were found in age, sex, or most vital signs among the groups. In contrast, weight, BMI, SBP, HR, RR, and several laboratory parameters showed significant group differences.

**Table 2:** Baseline characteristics of DKD patients stratified by serum lactate levels.

Variables	<2.0(*n* = 395)	2-4(*n* = 159)	≥4(*n* = 39)	total(*n*=593)	*P*
**Demographics**					
**Gender**					
Male (%)	273 (69.1%)	110 (69.2%)	22(56.4%)	405 (68.3%)	0.256
Female (%)	122 (30.9%)	49(30.8%)	17(43.6%)	188 (31.7%)	
Age (years)	72 (65, 79)	72 (65, 81)	78 (69, 83)	72 (65, 80)	0.088
Height (cm)	173(165, 178)	173 (163, 178)	168 (160, 178)	172 (163, 178)	0.658
Weight (kg)	88.4 (74, 104)	89 (75, 108)	77 (67, 90)	88 (73, 104)	0.005
BMI (kg/m²)	30.1 (26.3, 34.6)	31.1 (26.4, 36.8)	28.2 (24.0, 30.5)	30.1 (26.2, 35.1)	0.009
**Vital signs**					
SBP (mmHg)	119 (106, 137)	118 (103, 134)	113 (97, 128)	118(104, 135)	0.049
DBP (mmHg)	59 (52, 71)	61 (53, 71)	62 (53, 72)	60 (52, 71)	0.507
MAP (mmHg)	78 (69, 90)	78 (70, 91)	77 (68, 88)	78 (69, 89)	0.85
TP (°C)	36.6 (36.4, 37.0)	36.6 (36.3, 36.9)	36.9 (36.4, 37.2)	36.6 (36.4, 37.0)	0.244
HR (beats/min)	80 (70, 90)	87 (76, 99)	94 (82, 111)	81 (72, 94)	<0.001
RR (breaths/min)	18 (15, 22)	19 (16, 25)	20 (18, 24)	18 (16, 23)	<0.001
**Laboratory parameters**					
Lactate (mmol/L)	1.3 (1, 1.6)	2.5 (2.2, 2.9)	5.2 (4.4, 6.2)	1.6 (1.2, 2.4)	<0.001
Scr (mg/dL)	1.6 (1.2, 2.3)	1.5 (1.2, 2.5)	2.2 (1.7, 3.2)	1.6 (1.2, 2.4)	0.004
BUN (mg/dL)	31 (22, 48)	31 (19, 49)	39 (28, 62)	31 (22, 49)	0.024
SpO_2_ (%)	99 (96, 100)	99 (96, 100)	100 (95, 100)	99 (96, 100)	0.412
pH	7.36 (7.31, 7.40)	7.35 (7.31, 7.39)	7.30 (7.24, 7.36)	7.35 (7.31, 7.40)	<0.001
WBC (10^9^/L)	10.65 (7.8, 14.9)	13.5 (9.8, 17.8)	13.3 (6.7, 18.3)	11.3 (8.3, 16.0)	<0.001
RBC (10¹²/L)	3.37 (2.88, 3.84)	3.53 (3.03, 3.92)	3.46 (3.05, 3.95)	3.39 (2.93, 3.87)	0.177
PLT (10^9^/L)	180 (140, 238)	184 (138, 264)	192 (134, 248)	181 (139, 242)	0.727
HB (g/dL)	10.8 (9.2, 12.3)	11.1 (9.6, 12.5)	10.1 (8.8, 11.5)	10.9 (9.3, 12.3)	0.079
GLU (mg/dL)	158 (116, 219)	193 (151, 276)	209 (174, 315)	172 (126, 247)	<0.001
K⁺ (mmol/L)	4.2 (3.9, 4.7)	4.3 (3.9, 4.9)	4.0 (3.8, 5.3)	4.2 (3.9, 4.8)	0.307
Na⁺ (mmol/L)	138 (136, 141)	138 (136, 140)	137 (132, 142)	138 (136, 141)	0.675
HCO_3_^−^ (mmol/L)	22 (19, 24)	21 (19, 23)	17 (15, 20)	21 (19, 24)	<0.001
**Clinical outcomes and illness severity**					
Incidence of AKI (%)	335 (85%)	135(85%)	37(95%)	507(86%)	0.228
GCS score	14.30(2.41)	13.87(3.15)	14.67(1.15)	14.19(2.61)	0.209
SOFA score	2(1, 4)	3(2, 5)	8(4, 8)	3(1, 5)	<0.001
Sepsis (%)	228 (57.7%)	107 (67.3%)	32 (82.1%)	367(61.9%)	0.003
28-d mortality rate (%)	55(14%)	30(19%)	8(21%)	93(16%)	0.243
365-d mortality rate (%)	116(29%)	56(35%)	18(46%)	190(32%)	0.061

Data are presented as median (interquartile range) or number (percentage). BMI, body mass index; SBP, systolic blood pressure; DBP, diastolic blood pressure; MAP, mean arterial pressure; TP, body temperature; SpO_2_, peripheral oxygen saturation; Scr, serum creatinine; BUN, blood urea nitrogen; WBC, white blood cell count; RBC, red blood cell count; PLT, platelet count; HB, hemoglobin; GLU, glucose; K ⁺ , potassium ion; Na ⁺ , sodium ion; HCO_3_^−^, bicarbonate; AKI, acute kidney injury; GCS, Glasgow Coma Scale; SOFA, Sequential Organ Failure Assessment.

#### Association between blood lactate and Scr levels.

Multiple linear regression was used to examine whether lactate was independently associated with Scr (Table 3). In the unadjusted model (Model 1), each unit increase in lactate is associated with a significant increase in Scr (*β* = 0.093, 95% *CI*: 0.001–0.185, *P* = 0.048). This association remains statistically significant after sequential adjustments: following adjustment for sex, age, and BMI (Model 2; *β* = 0.104, 95% *CI*: 0.012–0.195, *P* = 0.027) and further adjustment for mean arterial pressure, oxygen saturation, and hemoglobin (Model 3; *β* = 0.095, 95% *CI*: 0.007–0.183, *P* = 0.034). In addition, after further adjustment for illness severity using SOFA and GCS (Model 4; complete-case N = 509), lactate remained independently associated with Scr (*β* = 0.130, 95% CI: 0.004–0.256; *P* = 0.044). A clinically targeted sensitivity analysis yielded similar results after further accounting for sepsis, illness severity, hemodynamic status, oxygenation, and hemoglobin (N = 409; β = 0.138, 95% CI: 0.006–0.270, P = 0.041; S3 Table).

**Table 3:** Association between serum lactate and serum creatinine in the MIMIC-IV cohort.

Model	β	95% CI	*P* value
**Model 1**	0.093	0.001–0.185	0.048
**Model 2**	0.104	0.012–0.195	0.027
**Model 3**	0.095	0.007–0.183	0.034
**Model 4**	0.130	0.004–0.256	0.044

*CI*: Confidence Interval. β: unstandardized regression coefficient.

Model 1: Unadjusted model.

Model 2: Adjusted for sex, age, and BMI.

Model 3: Adjusted for sex, age, BMI, MAP, SpO_2_, and hemoglobin.

Model 4: Adjusted for SOFA score and GCS score.

#### Subgroup analysis.

Using the fully adjusted Model 3, subgroup analyses were performed to further evaluate the robustness of the association between lactate and Scr across different patient characteristics (Table 4). Overall, the direction of the association remained generally consistent across most subgroups, although statistical significance was not observed in all strata. A significant interaction was identified for sex (*P* for interaction = 0.004), with a stronger positive lactate–Scr association observed in male patients than in female patients. This sex-related difference may partly reflect differences in muscle mass, creatinine generation, hormonal background, and metabolic or hemodynamic responses to critical illness; however, this subgroup finding should be interpreted as exploratory. In contrast, no significant interaction was observed for age or BMI, suggesting that the association was generally stable across age and BMI categories.

**Table 4:** Subgroup and interaction analyses of the association between serum lactate and Scr.

Variable	n (%)	β (95% *CI*)	*P*	*P* for interaction
**Total**	593 (100.00%)	0.095 (0.008 ~ 0.183)	0.034	
**Sex**				0.004
**Female**	188 (31.7%)	−0.070 (−0.230 ~ 0.088)	0.385	
**Male**	405 (68.3%)	0.192 (0.088 ~ 0.296)	<0.001	
**Age (year)**				0.881
**< 65**	141 (23.8%)	0.098 (-0.120 ~ 0.317)	0.378	
**≥ 65**	452 (76.2%)	0.103 (0.010 ~ 0.197)	0.031	
**BMI (kg/m²)**				0.205
**< 25**	86 (14.5%)	0.192 (-0.062 ~ 0.445)	0.136	
**≥ 25**	507 (85.5%)	0.066 (-0.028 ~ 0.16)	0.168	

#### External validation in the eICU cohort.

To evaluate the robustness and generalizability of the association between lactate and renal function observed in the MIMIC-IV cohort, an external validation analysis was conducted using the eICU Collaborative Research Database, a large multicenter critical care database in the United States. After applying inclusion and exclusion criteria consistent with those used in the MIMIC-IV cohort, 320 patients with DKD were retained for the validation analysis. Using the same exposure definition, renal outcome, and hierarchical regression modeling strategy, lactate remained positively associated with serum creatinine across all models.

In the unadjusted model (Model 1), lactate was significantly associated with higher serum creatinine levels (β = 0.162, 95% CI: 0.014–0.310, *P* = 0.031). After adjustment for age, sex, and BMI (Model 2), the association remained significant (β = 0.203, 95% CI: 0.062–0.345, *P* = 0.005). Further adjustment for mean arterial pressure, oxygen saturation, and hemoglobin (Model 3) strengthened the association (β = 0.247, 95% CI: 0.105–0.388, *P* = 0.001). After additional adjustment for illness severity using SOFA and GCS (Model 4), lactate remained independently associated with serum creatinine (β = 0.241, 95% CI: 0.098–0.384, *P* = 0.001) (Table 5). In the parallel clinically targeted sensitivity analysis, further adjustment for sepsis status did not materially alter the positive association between serum lactate and serum creatinine (N = 320; β = 0.269, 95% CI: 0.127–0.411, *P* < 0.001; S4 Table).

**Table 5:** External validation of the association between serum lactate and serum creatinine in the eICU cohort.

Model	N	β	95% CI	*P* value
Model 1	320	0.162	0.014–0.310	0.031
Model 2	320	0.203	0.062–0.345	0.005
Model 3	320	0.247	0.105–0.388	0.001
Model 4	320	0.241	0.098–0.384	0.001

*CI*: Confidence Interval. β: unstandardized regression coefficient.

Model 1: Unadjusted model.

Model 2: Adjusted for sex, age, and BMI.

Model 3: Adjusted for sex, age, BMI, MAP, SpO_2_, and hemoglobin.

Model 4: Adjusted for SOFA score and GCS score.

These findings were consistent with those observed in the MIMIC-IV cohort and further supported the robustness of the association between elevated lactate levels and impaired renal function in patients with DKD.

### Mechanistic exploration: Integrated network pharmacology analysis of lactate in DKD-related renal injury

#### Collection of lactate structural identifiers and putative targets.

Structural data for lactate, including SMILES and InChI identifiers, were obtained from the PubChem database. Possible protein targets were predicted through STITCH, PharmMapper and the ChEMBL database. After duplicates were removed, 198 unique targets remained for downstream analysis.

#### Screening of DKD-related targets and shared candidate targets.

**Fig 5:**
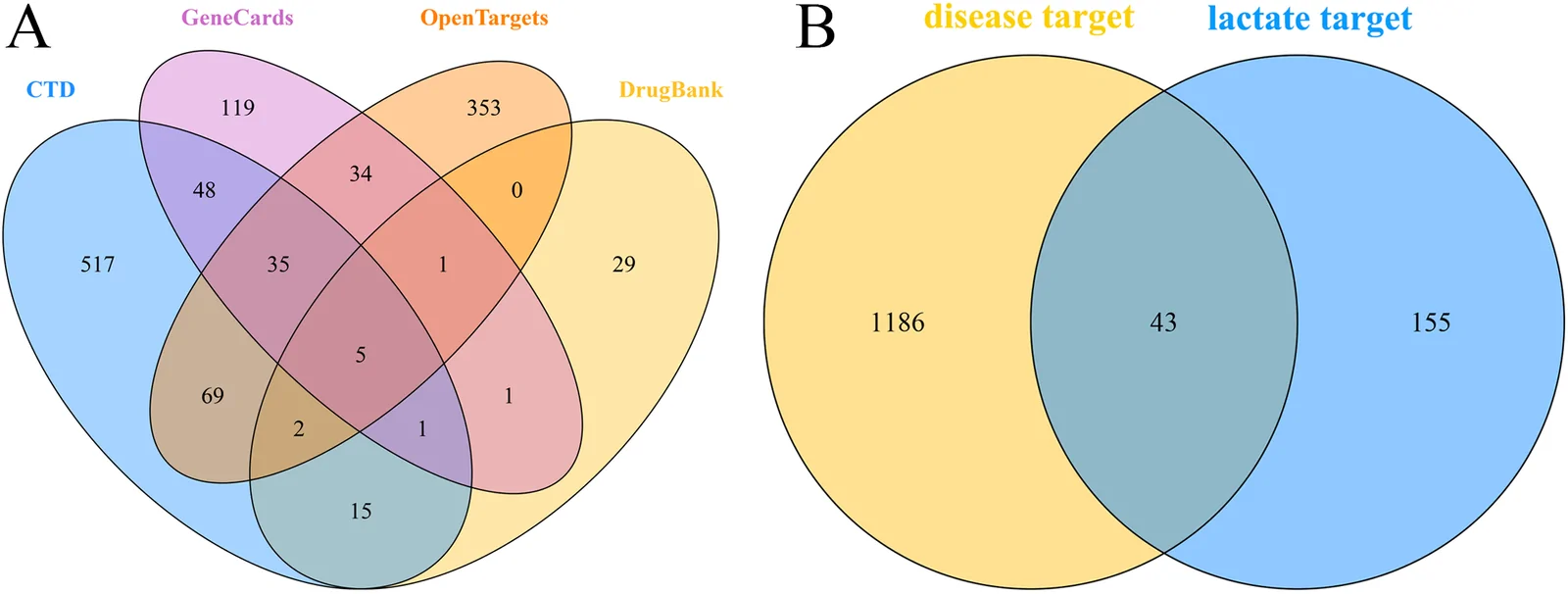
Identification of DKD-related targets and shared lactate–DKD candidate targets. (A) Venn diagram showing the overlap of DKD-related targets obtained from GeneCards, CTD, DrugBank, and Open Targets. (B) Venn diagram showing the intersection between lactate-related targets and DKD-related targets, yielding 43 shared candidate targets. The top 10 hub targets prioritized by PPI network degree centrality were PTGS2, SRC, EGFR, TP53, ESR1, MAPK3, MMP9, ADRB2, PTGS1, and PRKACA.

Disease-related targets are retrieved from the GeneCards (relevance score ≥ 20), CTD (inference score ≥ 40), DrugBank, and Open Targets (association score ≥ 0.05) databases. After standardization and removal of duplicates, a total of 1229 potential DKD-related targets are identified. The intersection between these disease targets and the 198 targets associated with lactate is analyzed, yielding 43 common targets that are considered as potential putative shared targets through which lactate may exert its effects on DKD. The distribution of DKD-related targets obtained from different databases and the overlap between lactate-related targets and DKD-related targets are shown in Fig 5A and 5B, respectively. A total of 43 shared targets were identified and further prioritized by PPI network topology analysis.

#### Construction and analysis of the lactate–target–disease network.

**Fig 6:**
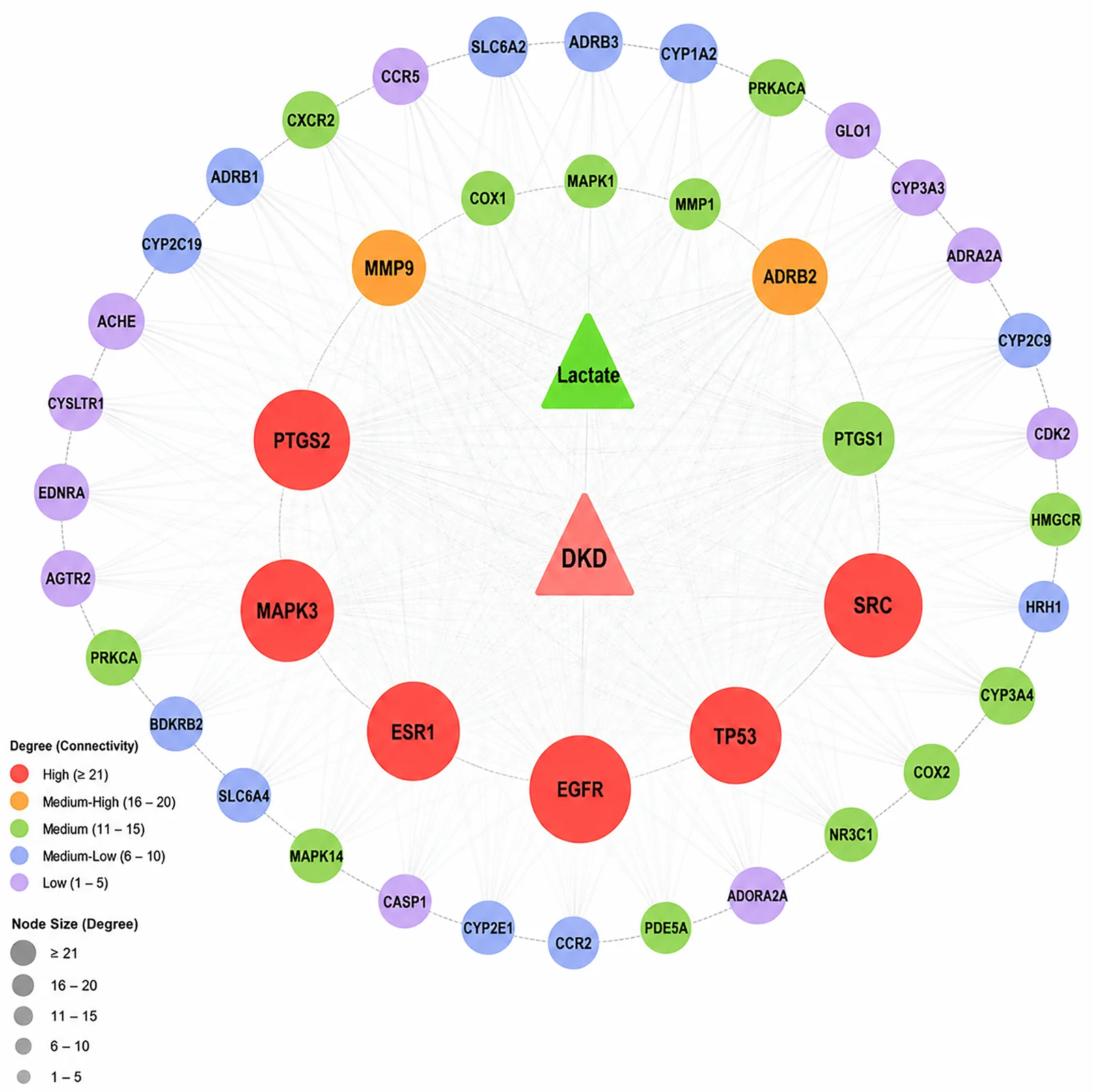
Lactate–target–disease interaction network. The network includes lactate, DKD, and the 43 shared lactate–DKD candidate targets. Target node size and color were scaled according to degree centrality derived from subsequent PPI network analysis. Larger nodes and warmer colors indicate higher degree values and greater topological priority. Edges represent database-derived lactate–target or target–DKD associations.

The lactate–target–disease interaction network, comprising lactate, the 43 shared targets, and DKD, is shown in Fig 6. In this network, lactate, DKD, and shared target proteins are displayed as distinct node categories. Target node size and color were scaled according to degree centrality derived from subsequent PPI network analysis. Larger nodes and warmer colors indicate higher degree values and greater topological priority, allowing high-priority hub targets, including PTGS2, SRC, EGFR, TP53, ESR1, MAPK3, MMP9, ADRB2, PTGS1, and PRKACA, to be visually identified. Edges represent database-derived lactate–target or target–disease associations and should be interpreted as putative associations rather than experimentally validated regulatory interactions.

#### Analysis of the PPI network.

**Fig 7:**
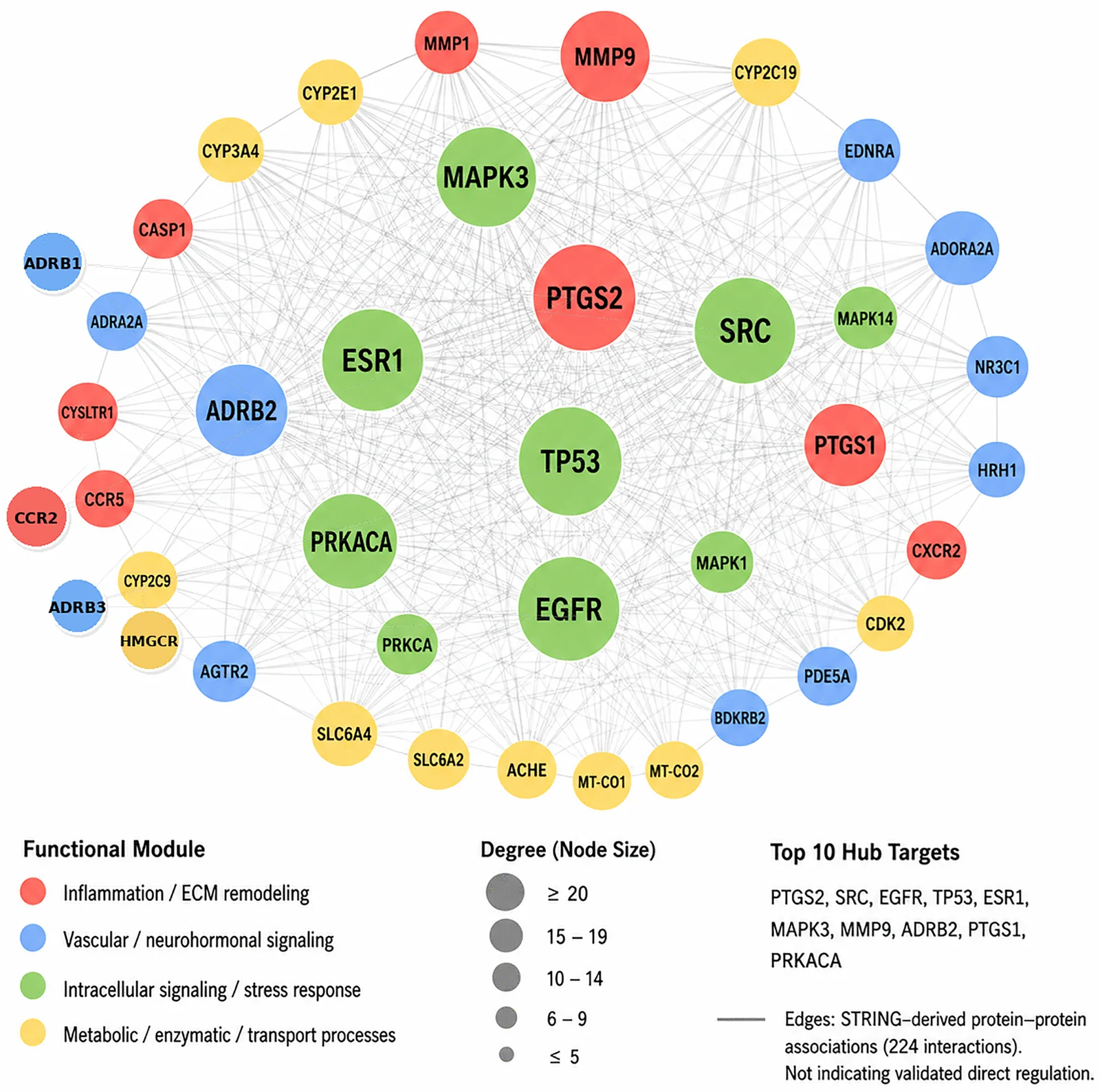
Module-oriented PPI network of shared lactate–DKD candidate target proteins.

**Fig 8:**
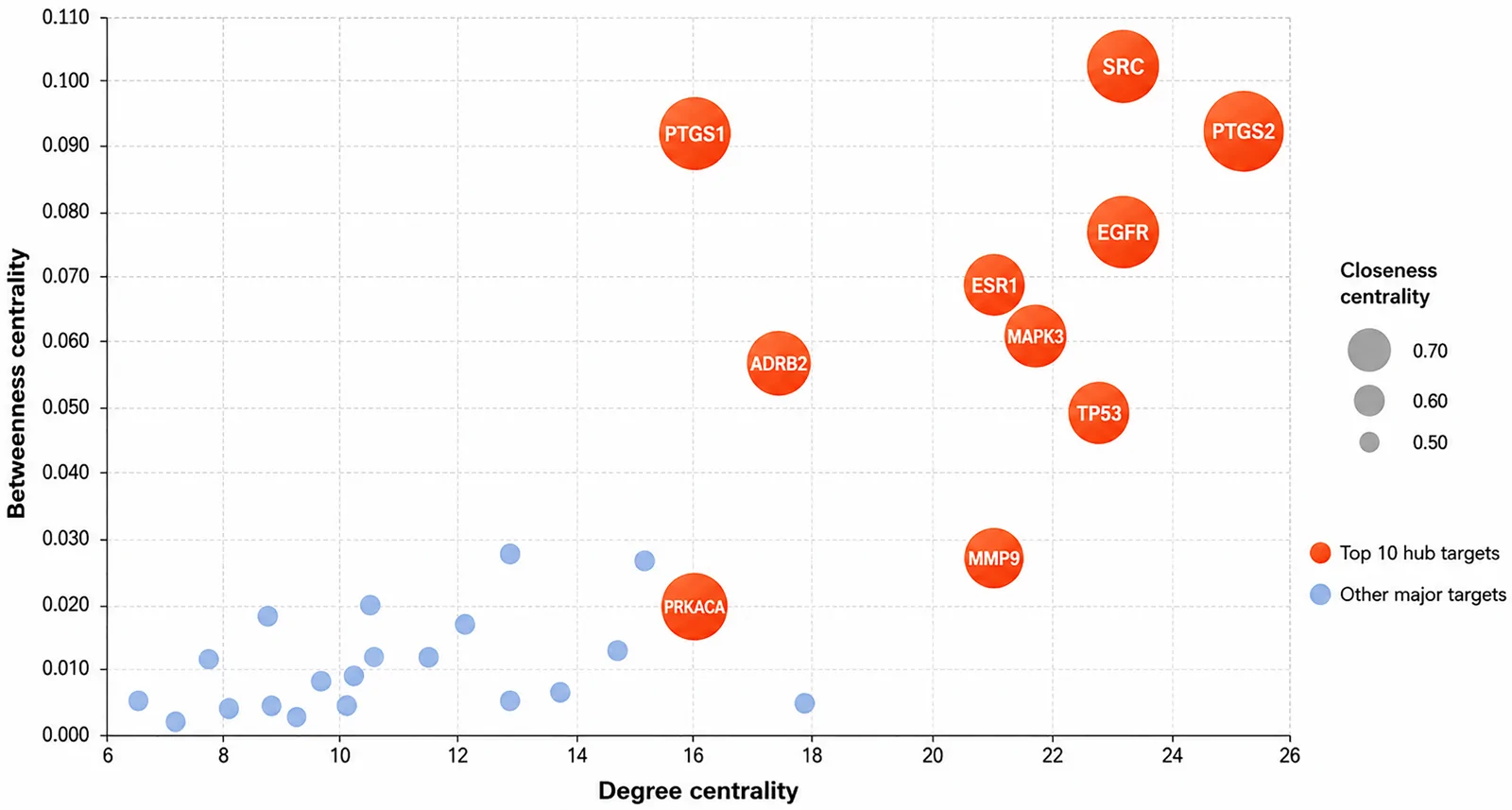
Integrated topological analysis of major PPI hub targets.

The 43 shared lactate–DKD targets were imported into STRING to construct a PPI network. After isolated nodes were excluded, the final PPI network contained 41 nodes and 224 interactions, representing STRING-derived functional or physical associations among the target proteins (Fig 7). To reduce redundancy and improve interpretability, the original STRING output and Cytoscape visualization were consolidated into a single module-oriented PPI network. In this network, nodes were organized according to functional annotations, including inflammation/ECM remodeling, vascular/neurohormonal signaling, intracellular signaling/stress response, and metabolic/enzymatic or transport-related processes. Node size was scaled according to degree centrality, allowing highly connected hub targets such as PTGS2, SRC, EGFR, TP53, ESR1, MAPK3, MMP9, ADRB2, PTGS1, and PRKACA to be visually prioritized. To strengthen hub-target prioritization, major hub genes were further evaluated by integrating degree centrality, betweenness centrality, and closeness centrality (Fig 8). In this integrated topology plot, degree centrality reflects local connectivity, betweenness centrality reflects the extent to which a node bridges different parts of the network, and closeness centrality reflects the relative proximity of a node to other nodes in the network. Hub targets such as PTGS2, SRC, EGFR, TP53, ESR1, MAPK3, MMP9, ADRB2, PTGS1, and PRKACA showed high values across one or more topological dimensions, supporting their prioritization as candidate network hubs rather than targets selected solely by degree centrality.

#### GO and KEGG enrichment analysis.

**Fig 9:**
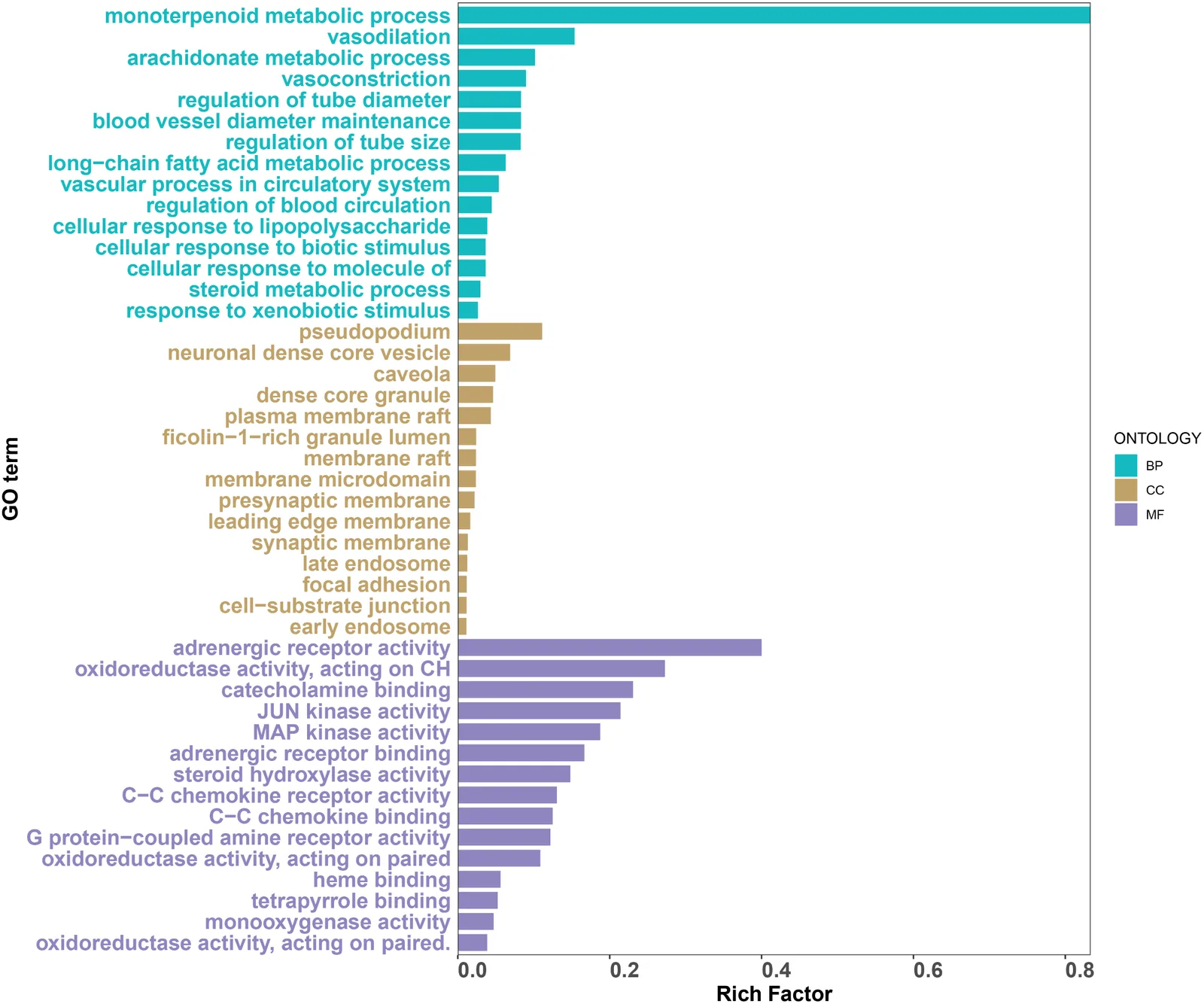
The Gene Ontology enrichment analysis identifies the top biological processes, cellular components, and molecular functions associated with the shared lactate-DKD targets.

**Fig 10:**
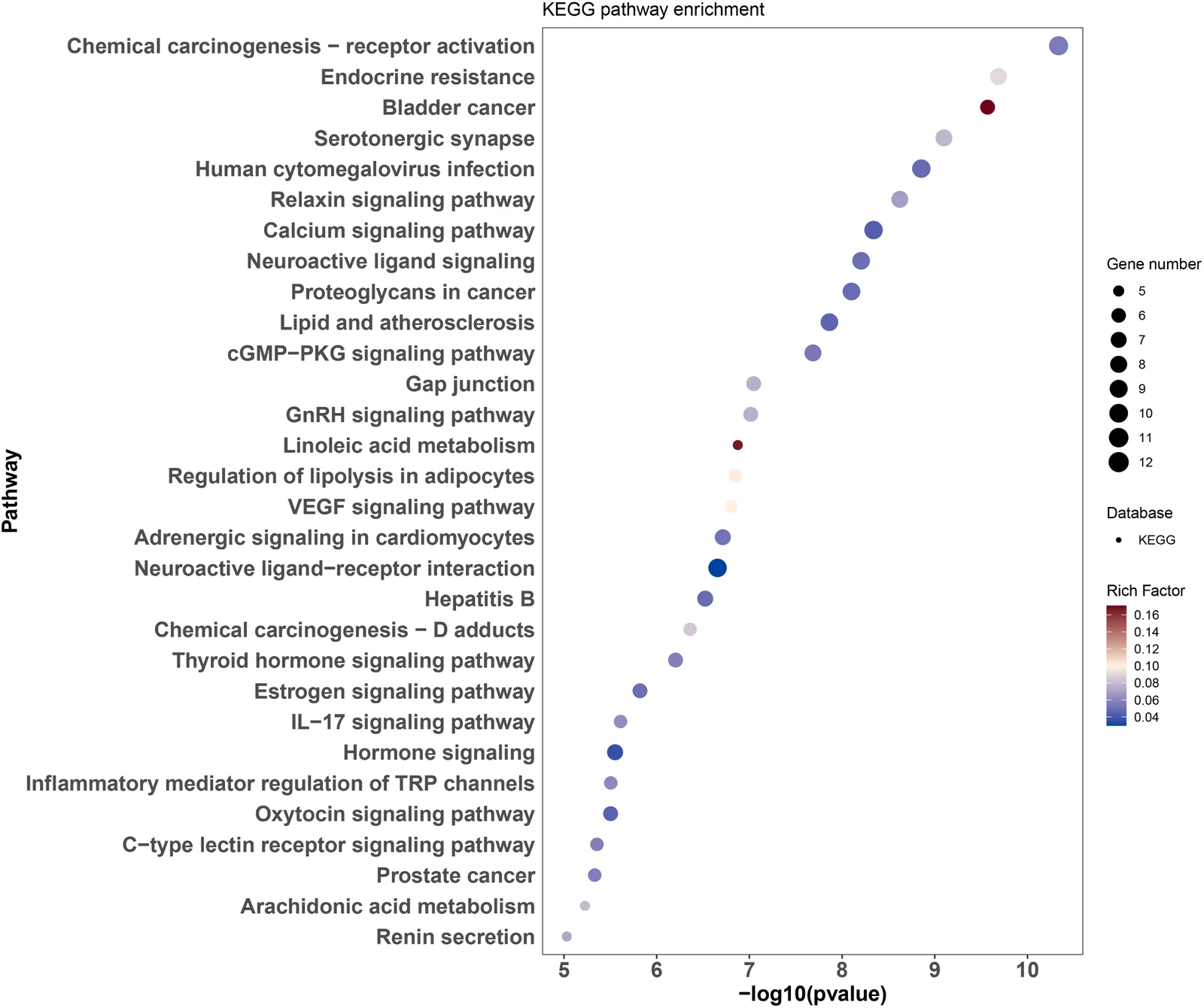
The KEGG pathway analysis highlights major signaling pathways enriched among the overlapping targets.

GO and KEGG enrichment analyses were conducted on the 43 shared targets using the R package *clusterProfiler*. The top 15 significantly enriched GO terms (*P* < 0.05) across biological process (BP), molecular function (MF), and cellular component (CC) categories are displayed in Fig 9. Key BP terms were mainly related to vascular regulation and lipid metabolic processes. CC terms were enriched in membrane microdomains and cell structure/adhesion-related components. MF terms were primarily associated with receptor functions and signaling/enzyme activities, including adrenergic/chemokine receptor activity as well as kinase- and oxidoreductase-related functions. KEGG enrichment revealed 150 pathways with significant involvement, and the 30 most enriched ones are displayed in Fig 10. Most of these pathways were related to calcium signaling, cGMP–PKG signaling, VEGF and relaxin signaling, immune/inflammatory pathways, lipid-related pathways, and renin secretion, which are consistent with processes previously implicated in DKD, such as vascular dysfunction, metabolic dysregulation, and inflammatory signaling.

#### Lactate–target–pathway–disease network.

**Fig 11:**
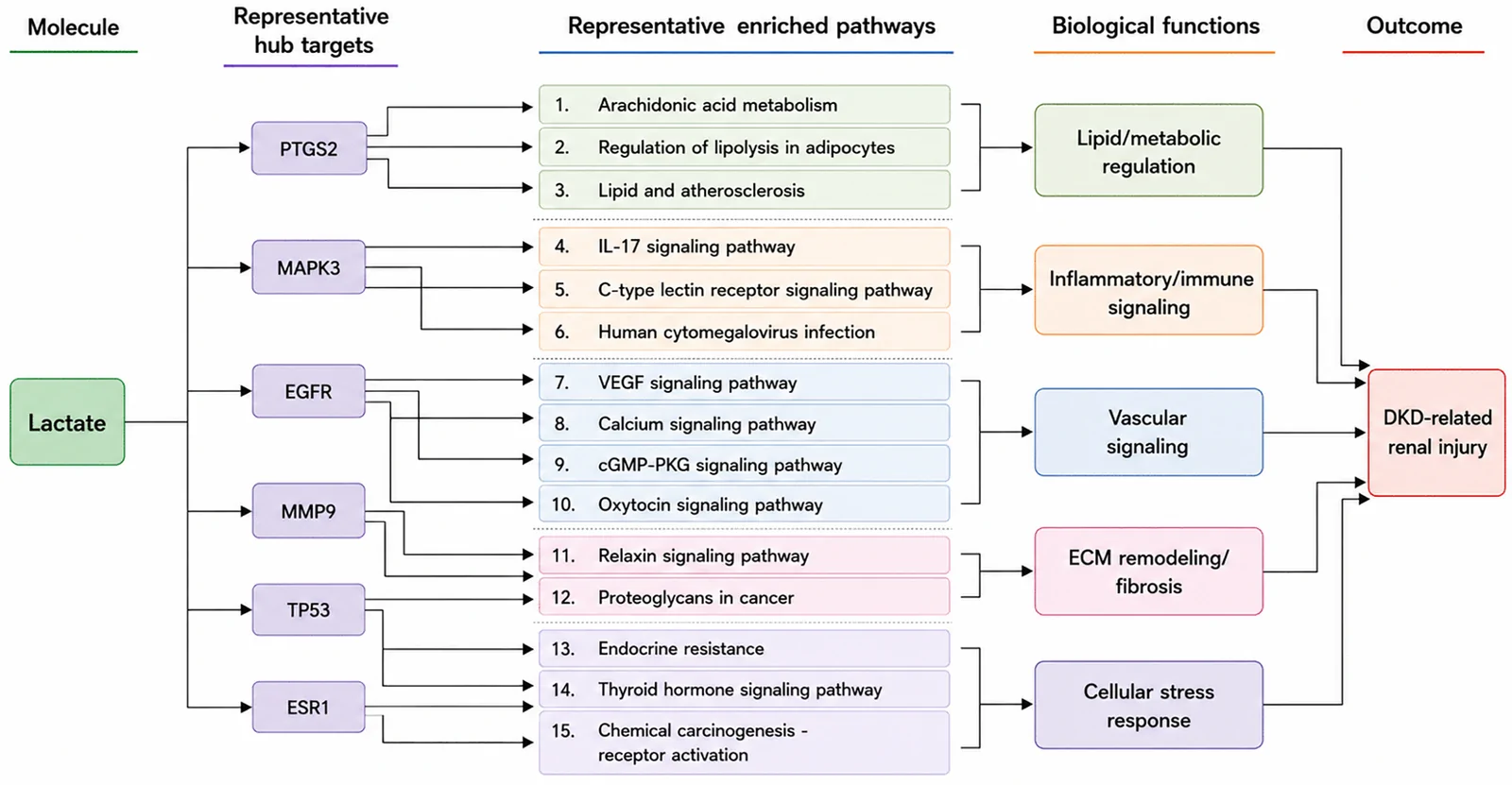
Hierarchical lactate–hub target–pathway–DKD network.

To improve interpretability, the lactate–target–pathway–DKD network was redesigned as a hierarchical layout (Fig 11). The revised network summarizes the putative progression from lactate to representative hub targets, enriched pathways, biological functions, and DKD-related renal injury. Representative hub targets included PTGS2, EGFR, TP53, ESR1, MAPK3, and MMP9. The enriched pathways were organized into major biological functions, including lipid/metabolic regulation, inflammatory/immune signaling, vascular signaling, ECM remodeling/fibrosis, and cellular stress response. This structured layout was used to summarize candidate molecular links rather than to infer sequential causality or experimentally validated regulation.

#### Molecular docking.

**Fig 12:**
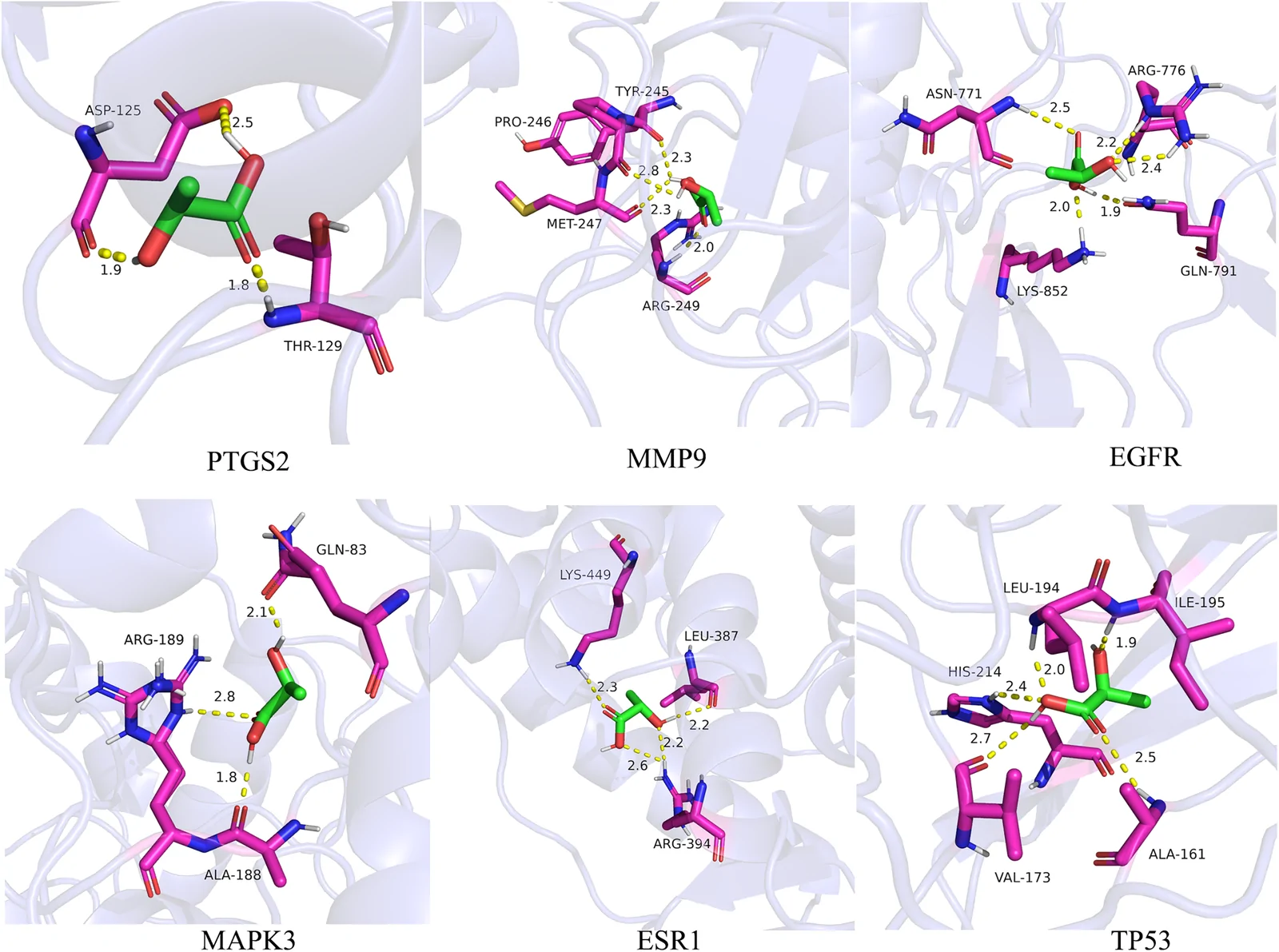
The schematic diagram shows the docking conformations of lactate within the active pockets of PTGS2, EGFR, TP53, ESR1, MAPK3, and MMP9.

To assess the plausibility of the predicted associations, six DKD-related core targets—PTGS2, EGFR, TP53, ESR1, MAPK3 and MMP9—were chosen as representative proteins for molecular docking with lactate (Fig 12). To achieve comprehensive biological coverage, these targets were selected from distinct functional modules identified in the network, rather than solely based on their topological ranking. Specifically, PTGS2 and MMP9 are closely linked to inflammatory signaling and extracellular matrix remodeling; EGFR and its downstream kinase MAPK3 are involved in key growth factor–related signaling; ESR1 reflects hormonal regulation and redox-related processes; and TP53 is a central stress-response regulator. The docking results indicated that lactate could be accommodated within the predicted binding pockets of all six proteins, with predicted binding energies ranging approximately from –3.8 to –4.9 kcal/mol. Among these, MAPK3 (–4.85 kcal/mol) and TP53 (–4.83 kcal/mol) showed the lowest predicted binding energies, followed by MMP9 (–4.73 kcal/mol), PTGS2 (–4.50 kcal/mol), ESR1 (–3.91 kcal/mol), and EGFR (–3.90 kcal/mol). Overall, the predicted binding energies suggest relatively weak to moderate interactions, which is compatible with lactate’s role as a small endogenous metabolite rather than a high-affinity inhibitor. These docking results suggest structural plausibility for lactate–protein associations with several candidate targets involved in fibrosis, oxidative stress, inflammation, and hormone signaling. However, these findings do not demonstrate biological activity or functional modulation and should be interpreted as hypothesis-generating.

## Discussion

### Summary of key findings

A notable discrepancy should be acknowledged: serum creatinine did not differ significantly in the rat model, whereas lactate was positively associated with serum creatinine in the MIMIC-IV and eICU cohorts. This difference may reflect the limited sensitivity of serum creatinine in a small exploratory animal experiment, particularly when renal injury is relatively early, partially compensated, or influenced by model-specific physiological adaptation after unilateral nephrectomy. In the animal model, Cys C, BUN, 24-h UTP, and histopathological changes may have captured renal injury more sensitively than Scr. By contrast, the clinical cohorts included older and critically ill DKD patients with broader variation in renal dysfunction, in whom serum creatinine was consistently available and clinically informative. Therefore, the translational interpretation of this study should be based on a broader pattern of lactate-associated renal injury or dysfunction rather than strict one-to-one concordance of Scr changes across animal and human datasets.

When we turned to the MIMIC-IV clinical dataset, the same trend appeared—patients with higher lactate tended to have worse renal outcomes. Even after adjusting for multiple variables, the relationship held, suggesting that the observed association between lactate accumulation and impaired kidney function is unlikely to be due to chance alone. Importantly, this association was further supported in an independent eICU validation cohort, in which lactate remained positively associated with serum creatinine across the same hierarchical models. In line with emerging literature linking lactate to metabolic stress and hypoxia-related signaling [6,19,20], our findings extend prior observations by providing an integrated experimental–clinical–computational framework that connects lactate-associated phenotypes to DKD-relevant pathways and candidate targets.

Importantly, the present study is primarily associative and hypothesis-generating in nature. Although consistent relationships between lactate levels and renal dysfunction were observed across experimental and clinical settings, causality cannot be inferred from these findings. Accordingly, lactate is best interpreted here as a metabolic marker and potential mediator of renal injury, rather than a confirmed causal driver of DKD progression.

Using a combined network pharmacology and molecular docking workflow, we identified around 43 possible protein targets that could connect lactate to DKD mechanisms. The protein–protein interaction and pathway analyses pointed to core networks related to inflammation, fibrosis, oxidative stress, and altered metabolism. Docking simulations showed that lactate could fit into several of these key proteins.

### From animal to human: Lactate as a potential biomarker and mediator in DKD

Across the animal and clinical datasets, elevated lactate was consistently observed in association with renal injury or dysfunction, although the specific renal markers differed between experimental and clinical settings. The parallel between the animal and clinical results supports the translational relevance of lactate-associated metabolic disturbance in DKD, but it does not establish lactate as a DKD-specific biomarker or causal mediator [21,22]. Rather, serum lactate should be interpreted as a non-specific metabolic indicator that may reflect renal dysfunction together with systemic metabolic and hemodynamic stress. Moreover, the positive association observed again in the external eICU cohort supports the robustness of this relationship across independent ICU databases, although the clinical interpretation should remain cautious because both cohorts were derived from critically ill populations. Taken together, these findings suggest that early lactate elevation may capture clinically relevant metabolic and hemodynamic stress associated with renal dysfunction in DKD. However, serum lactate is a non-specific metabolic indicator and may be influenced by infection or sepsis, tissue hypoperfusion, hypoxia, systemic stress, physical strain, medication exposure, substance-related factors, and dysfunction of organs involved in lactate production or clearance. These factors may interfere with its interpretation as a DKD-related biomarker, particularly in critically ill patients. Therefore, lactate should be interpreted as a marker of metabolic and hemodynamic stress associated with renal dysfunction rather than as a DKD-specific biomarker.

Clinically, serum lactate might work alongside traditional kidney markers such as creatinine and cystatin C, capturing shifts in redox balance and energy metabolism that appear before overt structural damage. A rise in lactate often mirrors increased glycolytic flow and mitochondrial strain, so tracking it could offer an early metabolic warning before irreversible injury develops.

That said, several caveats apply. The MIMIC-IV cohort mainly includes critically ill patients, which introduces selection bias and limits how far the findings extend to the broader DKD population [11]. Moreover, given the observational nature of the dataset, causality between lactate elevation and renal impairment cannot be established. Future studies combining experimental lactate modulation in animal and cellular models are warranted to verify whether altering lactate levels can directly affect DKD outcomes.

### Potential mechanisms: Molecular pathways associated with lactate

Network pharmacology and computational toxicology analyses suggest that lactate contributes to DKD through a *multi-target, multi-pathway* interaction network rather than a single signaling axis.

Within this network, EGFR and its downstream kinase MAPK3 (ERK1) represent a major signaling axis that has been implicated in diabetic renal injury and fibrotic remodeling. Sustained activation of the EGFR–ERK pathway has been reported to amplify profibrotic signaling (e.g., TGF-β–related responses) and extracellular matrix deposition, whereas pharmacologic EGFR inhibition can attenuate proteinuria and tubular injury in experimental DKD models [23–25]. MMP9 and PTGS2 (COX-2) are closely linked to inflammatory signaling and matrix remodeling. Experimental evidence suggests that MMP9 upregulation promotes leukocyte infiltration and extracellular-matrix turnover during renal injury, and that MMP9 inhibition can alleviate fibrotic progression [26]. In parallel, PTGS2 expression is increased in diabetic kidney injury, and COX-2 inhibition has been associated with reduced proteinuria and fibrosis in experimental settings [27]. While COX-2 participates in physiological renal homeostasis, persistent activation may shift toward pro-inflammatory and profibrotic signaling under chronic stress conditions. In contrast, ESR1 is associated with hormonal regulation and redox-related processes that may modulate susceptibility to metabolic stress. ESR1 signaling has been reported to support mitochondrial function and limit oxidative stress, potentially buffering injury responses in diabetic contexts [28]. TP53 links metabolic stress to cell-fate regulation. Recent studies in CKD have reported sustained activation of p53-related stress pathways in injured tubular cells, which is associated with apoptosis and impaired repair, suggesting that metabolic stressors may converge on this regulatory axis [29].Collectively, these molecular nodes form an integrated framework in which metabolic stress–related pathways involved in inflammation, oxidative stress, and fibrosis converge in DKD. Importantly, the network-based and docking findings in this study are hypothesis-generating; further mechanistic experiments are required to determine whether and how lactate modulates these targets in the diabetic kidney. Tissue-level molecular validation of these predicted hub targets was not performed in the present study; therefore, PTGS2, EGFR, TP53, ESR1, MAPK3, and MMP9 should be interpreted as prioritized candidate targets rather than experimentally validated mechanisms.

KEGG enrichment analysis further supported the network findings by highlighting pathways related to signal transduction and vascular–inflammatory regulation, including calcium signaling, cGMP–PKG signaling, VEGF signaling, and relaxin signaling. In addition, several enriched pathways were linked to immune and inflammatory responses, such as IL-17 signaling, C-type lectin receptor signaling, and inflammatory mediator regulation of TRP channels, together with lipid-related pathways (e.g., arachidonic acid metabolism, linoleic acid metabolism, and lipid and atherosclerosis) and renin secretion. Collectively, these enriched categories are consistent with processes previously implicated in DKD, including vascular dysfunction, metabolic dysregulation, and inflammatory signaling, and provide a plausible pathway-level context for the lactate-associated targets identified in this study.

Experimental evidence is broadly consistent with the KEGG-enriched pathways identified in this study. Lactate has been reported to act as a metabolic signal capable of modulating stress-response programs, including HIF-1 signaling under certain contexts, thereby linking lactate accumulation to hypoxia-responsive and vascular-related pathways [30,31]. In kidney injury models, glycolysis-related interventions provide further support for a lactate–inflammation/fibrosis connection: proximal tubule–specific deletion or pharmacologic inhibition of PFKFB3 reduced kidney lactate levels and mitigated renal inflammation and fibrosis [32]. In addition, several KEGG hits in our analysis implicate inflammatory and ion-channel signaling (e.g., IL-17 signaling and TRP channel–related pathways), both of which have been mechanistically linked to diabetic kidney injury and fibrotic progression in experimental studies [33,34].

In summary, elevated lactate levels are associated with DKD-related metabolic stress and with molecular pathways involved in inflammation, oxidative injury, and fibrosis. Serving as both a metabolite and signaling mediator, lactate emerges as a potential biomarker and a candidate mechanistic node warranting further experimental validation, rather than a confirmed therapeutic target, for DKD intervention.

### Implications and limitations

This study integrates metabolic evidence with renal functional alterations, bridging experimental findings and clinical observations [22]. By integrating network pharmacology analyses, this study provides a systems-level view of how metabolic stress may be associated with kidney injury [35]. Nevertheless, several limitations should be acknowledged. First, although renal histopathological staining was added to strengthen the characterization of the DKD rat model, the animal experiment remained exploratory and had a limited sample size. Therefore, the animal findings should still be interpreted as complementary evidence rather than definitive mechanistic proof. Moreover, the unilateral nephrectomy plus STZ-induced diabetic rat model may not fully capture the heterogeneity and chronic course of human DKD. Unilateral nephrectomy itself may also influence systemic or renal lactate metabolism through compensatory renal adaptation, altered renal hemodynamics, or ischemic stress. Because separate unilateral nephrectomy-only and STZ-only groups were not included, the present animal design cannot distinguish the individual contributions of nephrectomy-related renal adaptation and diabetes-related metabolic disturbance to lactate elevation. Future work should validate these findings in additional DKD models that include unilateral nephrectomy-only and STZ-only comparison groups and further investigate the molecular mechanisms underlying lactate-associated renal injury.

Second, the clinical analyses were derived from the MIMIC-IV database, which overrepresents critically ill patients and may introduce selection bias, thereby limiting generalization to the overall DKD population. Although illness severity and sepsis-related confounding were partly addressed through multivariable adjustment and the clinically targeted sensitivity analysis, residual confounding cannot be fully excluded, particularly with respect to septic shock, vasopressor use, pre-admission metformin exposure, medication history, substance-related factors, physical strain before admission, unmeasured organ dysfunction, and hemodynamic instability. In addition, modest differences between patients included in and excluded from the MIMIC-IV complete-case sensitivity analysis suggest that some complete-case selection may have occurred; therefore, this sensitivity analysis should be interpreted cautiously. Explicitly coded septic shock was not uniformly available in the current analytic dataset for reliable stratified or exclusion analyses; therefore, residual confounding related to shock physiology could not be fully excluded. Accordingly, future studies should validate the association in non-ICU cohorts and prospective settings with better confounder capture. Third, lactate was measured only once after admission, and data on serial lactate measurements and lactate clearance were unavailable, preventing assessment of temporal dynamics. Lactate clearance, typically reflecting the decrease in lactate over the first 24 h, may provide more informative risk assessment than a single static admission value because it captures the dynamic balance between lactate production, tissue perfusion, metabolic recovery, and treatment response. Therefore, persistent lactate elevation or impaired lactate clearance may better reflect ongoing systemic stress and renal-risk burden in critically ill patients. As with most observational analyses, some departures from ideal linear-model assumptions may remain despite standard diagnostic checks; therefore, the regression findings should be interpreted as indicative of association rather than precise effect estimation. Similarly, AKI ascertainment relied on creatinine-based KDIGO criteria; urine output and RRT/CRRT criteria were not incorporated, which could have led to under-ascertainment. To address these limitations, future research should evaluate longitudinal lactate trajectories, including 24-h lactate clearance, and more complete AKI definitions where feasible.

Fourth, the network pharmacology and molecular docking analyses were exploratory and hypothesis-generating in nature. Tissue-level molecular validation of the predicted hub targets, such as RT-qPCR or immunohistochemistry, was not performed in the present study. Therefore, the identified targets and docking results should not be interpreted as experimentally validated mechanisms. Future studies should validate prioritized targets and pathways using renal tissue assays, cellular models, and controlled lactate-modulation experiments.

## Conclusion

In conclusion, our findings suggest that lactate may contribute to DKD-associated renal injury through multiple interconnected mechanisms involving inflammation, fibrosis, oxidative stress, and metabolic dysregulation. These findings highlight lactate as a potential biomarker and candidate metabolic mediator in DKD, rather than a confirmed therapeutic target. Future studies should validate these insights experimentally, for example by modulating lactate levels in DKD models to assess renal and molecular responses [36]. Single-cell and spatial omics approaches could also clarify cell-type–specific metabolic and stress-response patterns, advancing our understanding of the metabolic–epigenetic interplay in DKD [37]. Collectively, these efforts may pave the way toward metabolic monitoring and precision therapy strategies centered on lactate regulation.

## Supporting information

S1 Table Between-group effect sizes for animal outcomes. (DOCX)

S2 Table Missing Data Handling Summary. (DOCX)

S3 Table Assessment of complete-case selection and clinically targeted sensitivity analysis in the MIMIC-IV cohort. (DOCX)

S4 Table Clinically targeted sensitivity analysis for the association between serum lactate and serum creatinine in the eICU cohort. (DOCX)
