# Supplementary material for: Associations between serum lactate and renal dysfunction in diabetic kidney disease: An exploratory analysis integrating animal experiments, clinical cohorts, and bioinformatic approaches
Source: PLoS One. 2026 Sep 15;21(9):e0358359. doi: 10.1371/journal.pone.0358359 (PMC13577499; doi:10.1371/journal.pone.0358359)
Supplement: Supplementary file 1 [file pone.0358359.s001.docx]

| **Supplementary Table S1: Between-group effect sizes for animal outcomes** | | | | |  |
| --- | --- | --- | --- | --- | --- |
| **Median (Control)** | **Median (DKD model)** | **Hodges–Lehmann median difference (Model − Control)** | **P value†** |  |  |
| 7.6 | 13.15 | 5.55 | 0.005 |  |  |
| 42.5 | 39 | −3.50 | 0.331 |  |  |
| 13.65 | 54.22 | 40.57 | 0.0022 |  |  |
| 0.5895 | 1.697 | 1.1075 | 0.0022 |  |  |
| 1.155 | 2.05 | 0.895 | 0.0022 |  |  |
| **Effect sizes are reported as Hodges–Lehmann median differences to mphasize magnitude and uncertainty in the context of small sample sizes.** | | | | |  |
|  |  |  |  |  |  |
