# Supplementary material for: Associations between serum lactate and renal dysfunction in diabetic kidney disease: An exploratory analysis integrating animal experiments, clinical cohorts, and bioinformatic approaches
Source: PLoS One. 2026 Sep 15;21(9):e0358359. doi: 10.1371/journal.pone.0358359 (PMC13577499; doi:10.1371/journal.pone.0358359)
Supplement: Supplementary file 2 [file pone.0358359.s002.docx]

**S2 Table. Missing-data handling summary**

| **Variable** | **Missing Proportion (n, %)** | **Imputation Method** |
| --- | --- | --- |
| temperature | 61 (10.3%) | Multiple Imputation（FCS） |
| heart_rate | 42 (7.1%) | Multiple Imputation（FCS） |
| resp_rate | 42 (7.1%) | Multiple Imputation（FCS） |
| SBP | 42 (7.1%) | Multiple Imputation（FCS） |
| DBP | 42 (7.1%) | Multiple Imputation（FCS） |
| MBP | 42 (7.1%) | Multiple Imputation（FCS） |
| SpO_2_ | 43 (7.3%) | Multiple Imputation（FCS） |
| pH | 0 |  |
| lactate | 0 |  |
| WBC | 5 (0.8%) | Multiple Imputation（FCS） |
| PLT | 5 (0.8%) | Multiple Imputation（FCS） |
| RBC | 5 (0.8%) | Multiple Imputation（FCS） |
| Scr | 0 |  |
| BUN | 0 |  |
| Glu | 0 |  |
| K⁺ | 0 |  |
| Na⁺ | 0 |  |
| HCO₃⁻ | 0 |  |
| HB | 4 (0.7%) | Multiple Imputation（FCS） |
| SOFA | 76 (12.8%) | Not imputed; complete-case analysis |
| GCS | 84 (14.2%) | Not imputed; complete-case analysis |

FCS, fully conditional specification.
