# Supplementary material for: Associations between serum lactate and renal dysfunction in diabetic kidney disease: An exploratory analysis integrating animal experiments, clinical cohorts, and bioinformatic approaches
Source: PLoS One. 2026 Sep 15;21(9):e0358359. doi: 10.1371/journal.pone.0358359 (PMC13577499; doi:10.1371/journal.pone.0358359)
Supplement: Supplementary file 3 [file pone.0358359.s003.docx]

**S3 Table. Assessment of complete-case selection and clinically targeted sensitivity analysis in the MIMIC-IV cohort**

**Panel A. Comparison between patients included in and excluded from the complete-case sensitivity analysis**

| **Variable** | **Included patients** | **Excluded patients** | **P value** |
| --- | --- | --- | --- |
| Serum lactate, mmol/L | 1.6 (1.2–2.1), n=409 | 1.6 (1.1–2.7), n=184 | 0.039 |
| Serum creatinine, mg/dL | 1.5 (1.2–2.3), n=409 | 1.7 (1.4–2.6), n=184 | 0.001 |
| SOFA score | 3 (1–5), n=409 | 2 (1–4), n=108 | 0.005 |
| GCS score | 15 (15–15), n=409 | 15 (14–15), n=100 | <0.001 |
| Sepsis, n (%) | 250 (61.1%), n=409 | 117 (63.6%), n=184 | 0.568 |

Note: Data in Panel A are presented as median (interquartile range) or number (percentage). Continuous variables were compared using the Mann–Whitney U test, and sepsis status was compared using the Pearson chi-square test. Variable-specific available sample sizes are reported because SOFA and GCS data were missing for some excluded patients.

**Panel B. Clinically targeted sensitivity analysis**

| **Analysis** | **N** | **β** | **95% CI** | ***P* value** |
| --- | --- | --- | --- | --- |
| Clinically targeted sensitivity model | 409 | 0.138 | 0.006–0.270 | 0.041 |

Note: In Panel B, β represents the unstandardized linear regression coefficient for the association between serum lactate and serum creatinine. The clinically targeted sensitivity model adjusted for age, sex, BMI, mean arterial pressure, oxygen saturation, hemoglobin, the first available SOFA score, the first available GCS score, and sepsis status. CI, confidence interval; BMI, body mass index; SOFA, Sequential Organ Failure Assessment; GCS, Glasgow Coma Scale.
