# Supplementary material for: Associations between serum lactate and renal dysfunction in diabetic kidney disease: An exploratory analysis integrating animal experiments, clinical cohorts, and bioinformatic approaches
Source: PLoS One. 2026 Sep 15;21(9):e0358359. doi: 10.1371/journal.pone.0358359 (PMC13577499; doi:10.1371/journal.pone.0358359)
Supplement: Supplementary file 4 [file pone.0358359.s004.docx]

**S4 Table. Clinically targeted sensitivity analysis for the association between serum lactate and serum creatinine in the eICU cohort**

| **Analysis** | **N** | **β** | **95% CI** | ***P* value** |
| --- | --- | --- | --- | --- |
| Clinically targeted sensitivity model | 320 | 0.269 | 0.127–0.411 | <0.001 |

Note: β represents the unstandardized linear regression coefficient for the association between serum lactate and serum creatinine. The clinically targeted sensitivity model adjusted for age, sex, BMI, mean arterial pressure, oxygen saturation, hemoglobin, SOFA score, GCS score, and sepsis status. CI, confidence interval; BMI, body mass index; SOFA, Sequential Organ Failure Assessment; GCS, Glasgow Coma Scale.
